# A genome-scale deep learning model to predict gene expression changes of genetic perturbations from multiplex biological networks

**DOI:** 10.1093/bib/bbae433

**Published:** 2024-09-03

**Authors:** Lingmin Zhan, Yingdong Wang, Aoyi Wang, Yuanyuan Zhang, Caiping Cheng, Jinzhong Zhao, Wuxia Zhang, Jianxin Chen, Peng Li

**Affiliations:** College of Basic Sciences, Shanxi Agricultural University, 1 Mingxian South Road, Taigu District, Jinzhong, 030801, China; College of Basic Sciences, Shanxi Agricultural University, 1 Mingxian South Road, Taigu District, Jinzhong, 030801, China; College of Basic Sciences, Shanxi Agricultural University, 1 Mingxian South Road, Taigu District, Jinzhong, 030801, China; College of Basic Sciences, Shanxi Agricultural University, 1 Mingxian South Road, Taigu District, Jinzhong, 030801, China; College of Basic Sciences, Shanxi Agricultural University, 1 Mingxian South Road, Taigu District, Jinzhong, 030801, China; College of Basic Sciences, Shanxi Agricultural University, 1 Mingxian South Road, Taigu District, Jinzhong, 030801, China; College of Basic Sciences, Shanxi Agricultural University, 1 Mingxian South Road, Taigu District, Jinzhong, 030801, China; School of Traditional Chinese Medicine, Beijing University of Chinese Medicine, 11 North Third Ring Road East, Chaoyang District, Beijing 100029, China; College of Basic Sciences, Shanxi Agricultural University, 1 Mingxian South Road, Taigu District, Jinzhong, 030801, China

**Keywords:** transcriptome, biological network, genetic perturbation, gene function, deep learning

## Abstract

Systematic characterization of biological effects to genetic perturbation is essential to the application of molecular biology and biomedicine. However, the experimental exhaustion of genetic perturbations on the genome-wide scale is challenging. Here, we show TranscriptionNet, a deep learning model that integrates multiple biological networks to systematically predict transcriptional profiles to three types of genetic perturbations based on transcriptional profiles induced by genetic perturbations in the L1000 project: RNA interference, clustered regularly interspaced short palindromic repeat, and overexpression. TranscriptionNet performs better than existing approaches in predicting inducible gene expression changes for all three types of genetic perturbations. TranscriptionNet can predict transcriptional profiles for all genes in existing biological networks and increases perturbational gene expression changes for each type of genetic perturbation from a few thousand to 26 945 genes. TranscriptionNet demonstrates strong generalization ability when comparing predicted and true gene expression changes on different external tasks. Overall, TranscriptionNet can systemically predict transcriptional consequences induced by perturbing genes on a genome-wide scale and thus holds promise to systemically detect gene function and enhance drug development and target discovery.

## Introduction

Functional characterization of genes is the core topic of life science, necessary to explore the genetic basis of biological traits, illustrate molecular mechanisms of diseases, and support new drug discovery and development [1–3]. In years past, both computational and experimental methods have been used to study gene function. The function of a gene or gene product can be inferred by mapping its sequence in the existing bioinformatic databases. The sequencing of the human genome has revealed a detailed catalog of genetic variation and mutations associated with many diseases [4].

However, the known gene–disease associations are insufficient to provide causal or mechanistic insights into uncharacterized genes; further genetic manipulation is required to directly interrogate gene function to understand how genes participate in biological molecular networks and lead to disease states, such as transgenic overexpression (OE), RNA interference (RNAi), and clustered regularly interspaced short palindromic repeat (CRISPR) loss-of-function technologies [5–8]. While these operations are often laborious, there is a lack of unified and comprehensive resources for systematically characterizing biological consequences of genetic perturbation at the genome scale. A wonderful solution is to create a catalog of cellular signatures representing the overall effects of perturbation of all genes in the genome. Following this concept, Connectivity Map (CMap) is developed as a public compendium of transcriptional responses of genetic perturbation and curates ~400 000 gene expression profiles induced by three types of genetic perturbations on various cell lines, including OE, RNAi, and CRISPR [9, 10]. This dataset provides opportunities to build functional connections between drugs, genes, and diseases at a gene expression level. Despite this, its small scale limits its utility. The newest CMap (updated in December 2020) contains genetic perturbations for only thousands of genes corresponding to OE, RNAi, and CRISPR respectively, which is much less than the number of genes (>20 000) across the whole human genome. This situation prompts us to establish a model for predicting inducible gene expression changes (GECs) by perturbing every gene in the genome, and this genome-scale resource can accelerate the characterization of gene function from a systematic level.

It has been known that different genes with similar features in biological networks function similarly [11, 12]. Therefore, in this work, we first produce integrated network features from various biological networks for each gene in the human genome. Then, we build a neural network model to map specific gene network features to GECs induced by corresponding genetic perturbations. In addition, we consider that there may be complementary information between GECs of three types of genetic perturbations for the same genes [13]; we improve the predicted GECs for one type of genetic perturbation by integrating the other two GECs for the same genes by a self-attention architecture.

## Results

### Problem formulation

Our task is to build a model to systemically predict transcriptional consequences induced by perturbing genes on a genome-wide scale. The transcriptional consequences adopt GECs of 978 landmark genes in the L1000 project, which has been verified to represent the reduced representation of cell transcriptome and can be used to infer the remainder of the transcriptional profile [10]. The genetic perturbations correspond to three classic genetic techniques, RNAi, CRISPR, and OE. Our first hypothesis is that the interference on different genes with similar biological functions causes similar biological consequences, including transcriptional profiles. Functional associations among genes are often represented as various gene–gene networks [14]. We can use these networks to learn the network-specific feature representation for each gene. The model is designed to map the gene representation to its GECs. The other assumption is that GECs induced by three types of genetic perturbations for the same gene have part-similar patterns in their inducible gene expression profiles. Thus, when predicting GECs of one type of genetic perturbation, the other two types of perturbations can supply complementary information for expression genes.

### TranscriptionNet architecture and training

**Figure 1:**
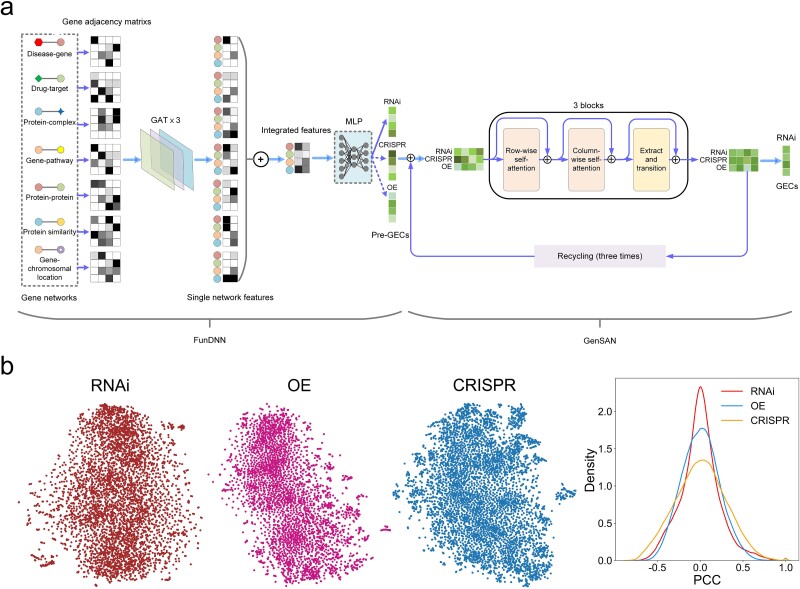
TranscriptionNet framework and distribution of GECs for RNAi, OE, and CRISPR. (a) The architecture of the TranscriptionNet model to predict GECs of RNAi. TranscriptionNet uses two-stage networks of FunDNN and GenSAN to load the genome-wide functional connection knowledge among genes and complementary information between different types of genetic interference manners on the same genes, respectively. FunDNN uses adjacency matrices for multiplex gene interaction networks as input. Each network passes through a graph attention network (GAT) to generate network-specific gene features, which are then combined into the integrated features. A stack of three GAT layers are used to generate gene features encompassing larger neighborhoods. The integrated features are propagated through multiple dense layers to fit pre-GECs induced by each type of genetic perturbation (RNAi, OE, and CRISPR). GenSAN takes GECs for all three types of genetic perturbations as input. To predict GECs for one type of genetic perturbation (e.g. RNAi), GenSAN processes the pre-GECs of RNAi and true GECs of CRISPR and OE together by a multilayer transformer encoder block (with axial self-attention in a dual-tower architecture) to capture complementary gene expression information between them. In the absence of known GECs for CRISPR or OE, the corresponding pre-GECs are used as alternatives. Moreover, the self-attention block is reinforced by feeding the processed GECs recursively into the same modules (named “recycling”). Finally, the predicted GECs for RNAi can be extracted from the output GEC matrix. (b) t-SNE analysis is performed on the GECs of the three genetic perturbations (RNAi, OE, and CRISPR), demonstrating the uniformity of data distribution. The distribution of PCCs among GECs of each genetic perturbation reveals the differences among different GECs.

The proposed TranscriptionNet adopts a two-stage coarse-to-fine network architecture, as shown in Fig. 1a. This coarse-to-fine network framework has been successfully used in image fields such as image inpainting and deblurring [15, 16], to improve the accuracy and generalizability of image processing. Inspired by this notion, we here take advantage of the two-stage networks to load the genome-wide functional connection knowledge among genes and complementary information between different types of genetic interference manners on the same genes, respectively. The first network that we name FunDNN (Functional network-based Deep Neural Network) is devoted to making gene functional representations and fitting the gene representation to its GECs induced by perturbing this gene. Specifically, FunDNN integrates various large-scale gene functional networks to encode a unified representation for each gene through a deep learning–based network integration algorithm, BIONIC (Biological Network Integration using Convolutions), which has been proved to perform better than existing network embedding methods on a range of benchmark tasks [17]. Each network-specific representation is then run through a sequence of fully connected layers to learn the first-stage GECs (termed pre-GECs) for each type of genetic perturbation. The second network that we term GenSAN (Genetic perturbation type–based Self-Attention Network) takes GECs for all three types of genetic perturbations as input. To predict GECs for one type of genetic perturbation (e.g. RNAi), GenSAN processes the pre-GECs of RNAi and true GECs of CRISPR and OE together by a multilayer transformer encoder block to capture complementary gene expression information between them. In the absence of known GECs for CRISPR or OE, the corresponding pre-GECs are used as alternatives. Moreover, the self-attention block is reinforced by feeding the processed GECs recursively into the same modules (named “recycling”). The pre-GECs internalizing complementary information from the other two types of genetic perturbations pass multiple dense layers to obtain final predictive GECs.

We use the latest high-throughput CMAP dataset from the L1000 platform to fit the model. The dataset contains GECs induced by three types of genetic perturbations on 8184 genes, corresponding to 4454 RNAi, 5139 CRISPR, and 3538 OE. Values of each gene are normalized with the MinMax scaler (see Methods). Analysis of GECs for each type of genetic perturbation using *t*-distributed stochastic neighbor embedding (t-SNE) shows that all types of data are distributed uniformly (Fig. 1b). As the big difference between GECs induced by the three types of genetic manipulation techniques [10, 13], each type is trained independently with similar model architecture but different hyperparameters. The input of FunDNN includes seven diverse human gene networks: the disease-based gene association network (995 genes, 4047 interactions), the drug-based gene association network (2792 genes, 131 193 interactions), the protein complex–based network (3407 genes, 40 170 interactions), the pathway-based gene network (10 623 genes, 1 787 207 interactions), the chromosomal location–based gene network (26 813 genes, 860 164 interactions), the Search Tool for Recurring Instances of Neighboring Genes (STRING) protein–protein interaction network (17 844 genes, 535 462 interactions), and the protein sequence similarity network (18 586 genes, 4 156 924 interactions), which combine for a total of 26 945 unique genes and 7 515 167 unique interactions (Supplementary Table 1). For GenSAN, it receives the pre-GECs for one type of genetic perturbation from the upstream FunDNN, combined with true GECs (or corresponding pre-GECs as alternatives in the absence of true GECs) for the other two types of genetic perturbations on the same gene. Both FunDNN and GenSAN use a customized reconstruction loss termed PMSE that combines the Pearson correlation and mean squared error (MSE) to minimize the discrepancy between predicted and true GECs.

### Evaluation strategies and metrics

For each type of genetic perturbation, all data are randomly split into training, validation, and test sets with a 7:1:2 ratio. The training set is used to fit the model, whose performance is evaluated by the hold-out test set. Pearson correlation coefficients (PCCs) are used as the major metric to evaluate the performance of models. We can quantify the correlation for each pair of predicted and true GECs and compare different models through the distribution or average of PCC values in the test set. In addition, MSE is used to assess the numerical difference between predicted and true GECs, and the Kolmogorov–Smirnov (KS) test statistic maximum distance (D) is used to compare the coherence of distribution of predicted and true GECs.

To further evaluate the generalization ability of TranscriptionNet, we predict GECs by TranscriptionNet for external genetic perturbations (corresponding to all network genes whose GECs are not profiled in the L1000 project) and compare their quality with known GECs in the L1000 project through the following analyses: (i) gene coannotation prediction; (ii) compound–target interaction prediction, and (iii) disease–gene association detection. First, we compare the performance of external and known GECs to identify gene pairs coannotated to the same functional term using a binary classification strategy in which gene pairs within at least one functional annotation are regarded as positive pairs, while gene pairs not within an annotation are retained as negative pairs (see Methods) [17]. Second, we compare the ability of external and known GECs to recover known compound–target interactions based simply on the similarity between GECs of genetic perturbations and compounds of targets. Finally, we compared the quality of external and known GECs, using the same binary classification strategy to identify genes associated with disease. The known disease-associated genes are considered to be positive, while genes not in the disease-associated gene set were retained as negative (see Methods). These external evaluations determine how effectively the predicted GECs can be used for additional tasks compared with existing GECs.

### Performance evaluation

**Figure 2:**
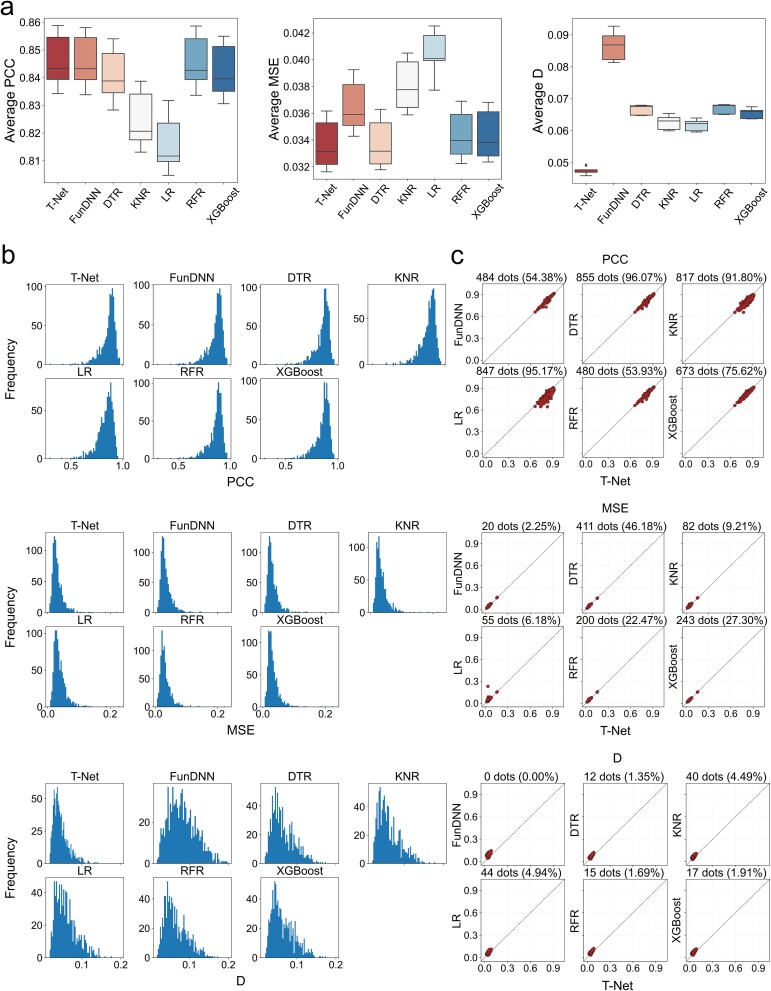
Comparison of TranscriptionNet to baseline models for predicting GECs of RNAi. (a) Box plots for three metrics (PCC, MSE, KS test statistic D) on the test dataset for seven models: TranscriptionNet, FunDNN, DTR, KNR, LR, RFR, and XGBoost. (b) Distribution of PCC, MSE, and D for the seven models on the test dataset. Data are obtained from five random runs. (c) The profile-wise comparative analysis of PCC, MSE, and D for each predicted GECs in the test set between TranscriptionNet and the other six models. The *x*-axis represents the results predicted by the TranscriptionNet model, while the *y*-axis represents the results predicted by the other six models. The dots below the diagonal indicate that TranscriptionNet has higher PCC values and lower MSE and D values compared to other models.

Prediction of GECs is a classical multivariate linear regression (MLR) problem in machine learning (ML). Here, we compare TranscriptionNet to four baseline regression algorithms under the random split setting: Decision Tree Regression (DTR), *K*-Nearest Neighbors Regression (KNR), Linear Regression (LR), Random Forest Regression (RFR), and eXtreme Gradient Boosting (XGBoost). The inputs of the four regressors are network-specific gene features derived from BIONIC, similar to FunDNN that can be regarded as a deep neural network regression algorithm. As shown in Fig. 2a, the GECs predicted by these regression algorithms are all well fitted with true GECs in the test set [PCC averages of 0.816–0.846 for RNAi, 0.822–0.843 for CRISPR (Supplementary Fig. 1), and 0.919–0.939 for OE (Supplementary Fig. 2)], convincing the reliability of mapping robust gene functional representations onto transcriptional responses of the corresponding genetic perturbations. Especially, for all three types of genetic perturbations, FunDNN performs better than the four ML regressors in terms of PCC averages, while its performance in MSE and D is also competitive. Furthermore, TranscriptionNet, which combines FunDNN and GenSAN, consistently outperforms baselines and individual FunDNN, with optimal PCC and D averages for all three types of genetic perturbations. We also compare the detailed distribution of all metrics between different models. TranscriptionNet also outperforms baselines and FunDNN for almost all of GECs (Fig. 2b). The profile-wise comparative analysis shows that the percentage of GECs predicted by TranscriptionNet with larger PCC outperforms FunDNN, DTR, KNR, LR, RFR, and XGBoost by 54.38%, 96.07%, 91.80%, 95.17%, 53.93%, and 75.62%, respectively (Fig. 2c). Additionally, statistical tests using the *T*-test are conducted to compare the performance among different models, and the statistical significance between these models is presented in Supplementary Table 4. The TranscriptionNet models for the three perturbation types of RNAi, OE, and CRISPR exhibit significant differences in PCC and MSE compared to the KNR and LR models and also demonstrated significant differences in D compared to all five classical MLR models. These results indicate that by importing correlation information between three types of genetic perturbations on the same gene, GenSAN can effectively improve the quality of pre-GECs predicted by FunDNN. We select 10 target genes for three types of genetic perturbations based on their ranking values of PCCs and detail the distribution between corresponding actual and predicted GEC data. We observe that the GECs from all three types of genetic perturbations are extremely well fitted (scatter plots are shown in Supplementary Figure 3). Network integration is the primary module for TranscriptionNet. An excellent network integration algorithm should produce accurate and comprehensive gene representations from biological networks. BIONIC used in our model has been proven to outperform existing integration methods across all evaluation types and benchmarks [17]. To further confirm the potency of BIONIC in our experiment, we replace BIONIC in TranscriptionNet with three different established integration approaches to compare their performance: a naive union of networks (Union), a deep learning multimodal autoencoder (deepNF) [18], and a multinetwork extension of the node2vec [19] model (multi-node2vec) [20]. We observe that BIONIC outperforms the established integration approaches in terms of PCC, MSE, and D (Supplementary Fig. 4). In addition, to ensure the advantage of gene features encoded from multiple networks over single networks, we compare the results of TranscriptionNet using multiple networks with those using single networks. As expected, gene features learned from multiple networks perform as well as, or better than, the individual input networks across all evaluation metrics (see the results for RNAi, OE, and CRISPR in Supplementary Figures 5–7, respectively). These results demonstrate the strength of BIONIC for encoding suitable gene representations in our model.

### Characterization of gene function

**Figure 3:**
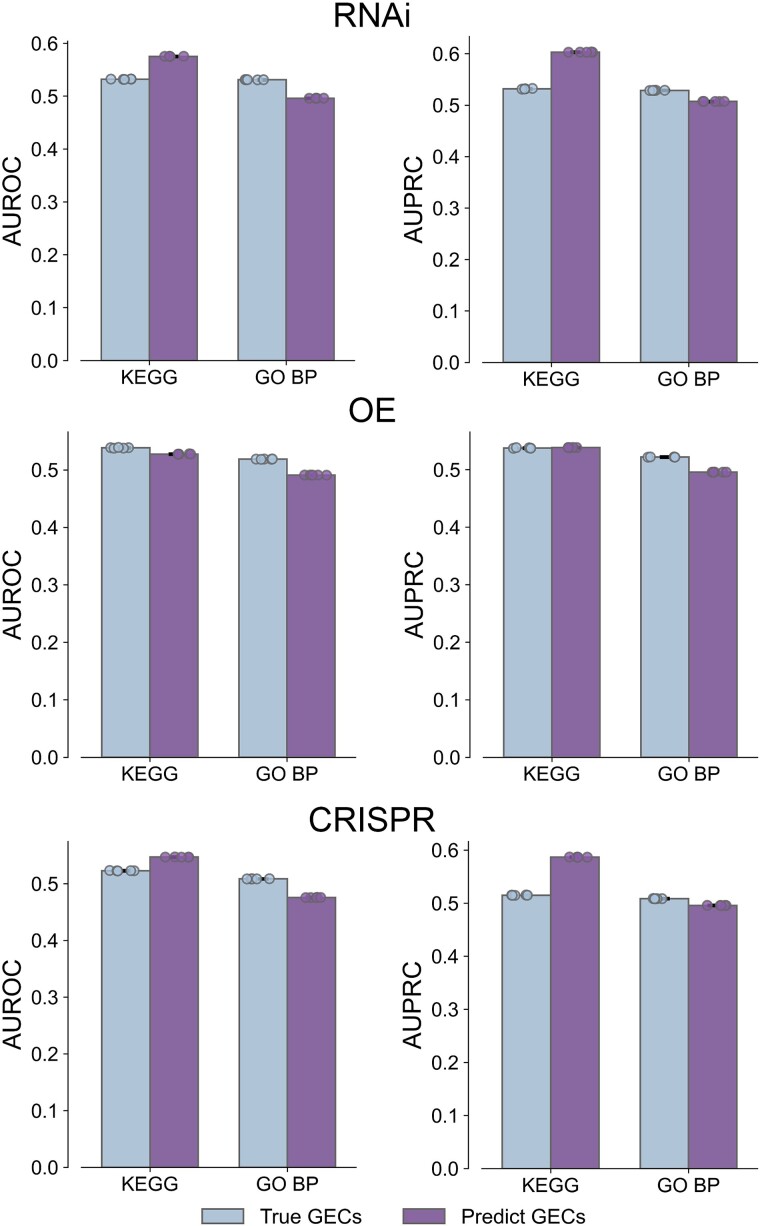
Gene function characterization by true and predicted GECs. The co-annotation prediction evaluations of true and predicted GECs in KEGG pathways and GO BP benchmarks using RNAi, OE, and CRISPR genetic perturbations. The statistics are carried out over five random runs. The evaluation criteria include the AUROC and AUPRC. The data are represented as the average value, with error bars indicating the 95% confidence interval of five independent samples and floating points representing the accurate values of five independent samples.

TranscriptionNet is used to predict GECs for external genetic perturbations corresponding to gene members in all input networks except perturbations profiled in the CMap dataset, resulting in GECs for 22 496 RNAi, 21 806 CRISPR, and 23 427 OE (https://github.com/lipi12q/TranscriptionNet/tree/master). To assess the quality of these predicted GECs, we compare the ability of predicted and known GECs to recover gene pairs coannotated to the same functional term. For all three types of genetic perturbations, the external GECs have similar performance to known GECs at identifying coannotated gene pairs with over two different functional benchmarks that are not used in our model: Kyoto Encyclopedia of Genes and Genomes (KEGG) pathways [21] and GO biological processes (BPs) [22] (Fig. 3). The similar performance between external and known GECs is obtained for coannotated analyses in the functional networks used in our model, including the disease-based gene association network, the drug-based gene association network, the protein complex–based network, the pathway-based gene network, the chromosomal location–based gene network, the STRING protein–protein interaction network, and the protein sequence similarity network (Supplementary Fig. 8). The similar performance of external and known GECs in the characterization of gene function verifies the high quality of GECs predicted by TranscriptionNet.

### Characterization of compound–target interactions

**Figure 4:**
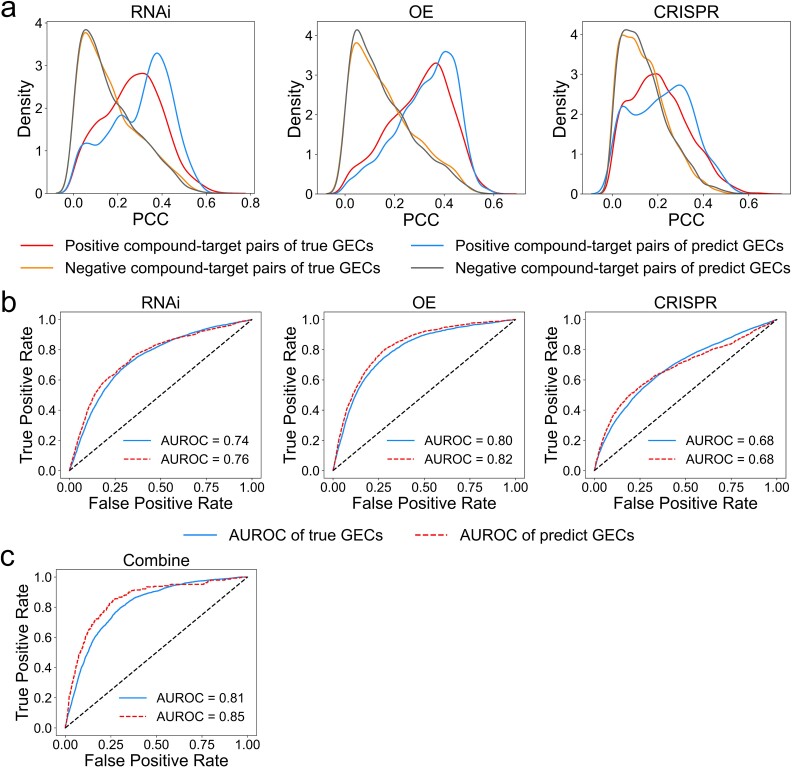
Characterization of compound–target interactions by true and predicted GECs. (a) The distribution of PCC between GECs in positive and negative compound–target pairs for true and predicted GECs. The distribution curves are separately plotted for RNAi, OE, and CRISPR perturbations. (b) ROC curves for predicting compound–target interactions by true and predicted GECs correlation for the three genetic perturbations: RNAi, OE, and CRISPR. (c) ROC curve of true and predicted GECs correlation by combining three types of genetic perturbations for the same genes using a logistic regression algorithm.

Theoretically, we can interrogate connections between drugs and protein targets simply through correlations of transcriptional profiles induced by drugs and genetic perturbations. To assess the feasibility of using predicted GECs in such an approach, we compare the ability to recover known drug–target interactions by predicted GECs and known GECs. We directly calculate PCCs between 33 609 compounds and 26 945 genetic perturbations (including known GECs and predicted GECs), resulting in 905 459 780 relationships. Among them, 8995 pairs have been curated in CMap as real drug–target interactions (Supplementary Data 1). We refer to these interactions as the positive set, while all remaining pairs are retained as the negative set. Firstly, for each type of genetic perturbation, we compare the distribution of correlations between drugs and targets with known and predicted GECs in positive and negative sets, respectively. As shown in Fig. 4a, we observe that whether for predicted or true GECs, drug–target pairs in the positive set are extremely more correlated than those in the negative set. Moreover, there is a similar distribution of drug–target correlations for true and predicted GECs in positive or negative sets.

We further quantify the performance of GECs for discriminating different types of drug–target pairs by a receiver operator characteristic (ROC) curve. We calculate the true positive rate and the false positive rate and plot ROC curves for known and predicted GECs based on various thresholds of correlation coefficients. As shown in Fig. 4b, we find both known and predicted GECs perform similarly and well for all three types of genetic perturbations [area under the receiver operating characteristic curve (AUROC) of 0.74 and 0.76 for RNAi, 0.80 and 0.82 for CRISPR, and all 0.68 for OE]. When correlations are summarized across all three types of perturbations by the logistic linear regression algorithm, the performance has a minor improvement with AUROC of 0.81 and 0.85 for known and predicted GECs, respectively (Fig. 4c).

These results indicate that the GECs predicted by TranscriptionNet are similar to known GECs and can be used to explore drug–target interactions.

### Characterization of disease–gene associations

**Figure 5:**
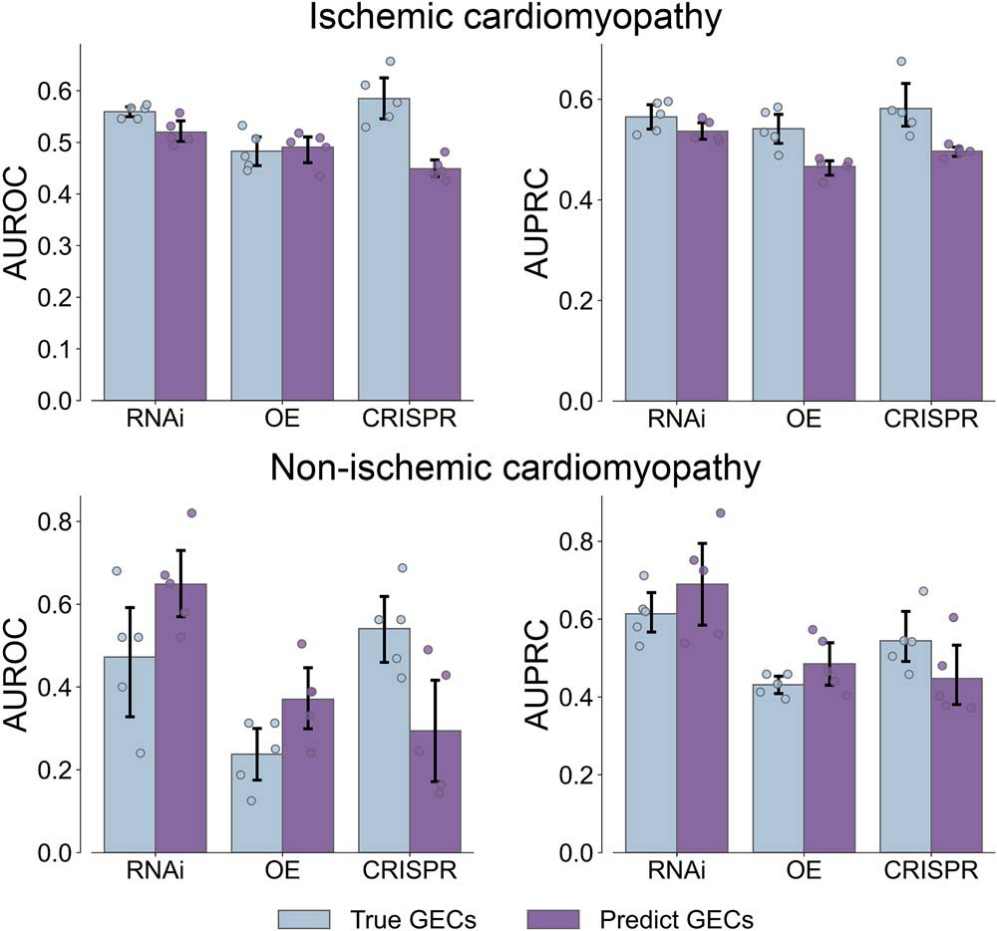
Characterizing associated genes for ischemic cardiomyopathy and nonischemic cardiomyopathy by true and predicted GECs. The performance of predicting disease–gene associations by directly calculating PCC between GECs induced by genes and GECs induced by ischemic cardiomyopathy and nonischemic cardiomyopathy. We separately analyze the three types of genetic perturbations: RNAi, OE, and CRISPR. The statistics are carried out over five random runs. The evaluation criteria include the AUROC and AUPRC. The data are represented as the average value, with error bars indicating the 95% confidence interval of five independent sample and floating points representing the accurate values of five independent samples.

To evaluate the feasibility of using predicted GECs to study the association between diseases and target genes, we compared the ability of predicted GECs and known GECs to recover known disease–gene associations. We here use the myocardial transcriptomic data for ischemic and nonischemic cardiomyopathy from the study of Yang *et al*. [23]. This work conducted deep sequencing of RNA isolated from paired nonischemic and ischemic human failing heart samples. The two diseases have many similarities from molecular to pathological phenotype and thus are hard to be distinguished. We attempt to compare the ability of predicted and known GECs to recover related genes for the two diseases. Based on the differential gene expression profiles induced by ischemic cardiomyopathy and nonischemic cardiomyopathy, we directly calculated the PCC between GECs for all 26 945 genetic perturbations (including known GECs and predicted GECs) and the differential expression profiles of the two diseases. The resulting gene lists ranked by PCC are separately mapped to known 110 and 15 genes associated with ischemic cardiomyopathy and nonischemic cardiomyopathy. Among them, the genes associated with cardiomyopathy are referred to as the positive set, while all other genes are retained as the negative set. The performance of GECs in distinguishing different types of disease–gene pairs is further quantified by AUROC and area under the precision–recall curve (AUPRC). We find that true and predicted GECs perform similarly across three genetic perturbations (Fig. 5). These results demonstrate that the GECs predicted by TranscriptionNet have a similar ability to known GECs to characterize disease–gene associations, confirming the quality of the GECs predicted by TranscriptionNet.

## Discussion

Systematic characterization of gene function can enhance the exploration of pathological mechanisms of diseases and aid in target–gene-based drug discovery. As transcriptional profiles represent the overall molecular activities of cells, genetic perturbation–induced transcriptional profiles are excellent molecular features to characterize the biological function of genes. Although techniques like RNA-array and high-throughput sequencing have been extremely applied for gene expression profiling, their genome-wide scalability is limited due to huge costs. TranscriptionNet can serve as a valuable complement to these experiments. We train TranscriptionNet based on transcriptional profiles associated with three different perturbations (RNAi, CRISPR, and OE) on thousands of genes that are generated by the L1000 project [10]. TranscriptionNet has been used to infer GECs of unknown genetic perturbations on the genome-wide scale and increases perturbational GECs from thousands of genes to 26 945 genes for each type of genetic perturbation. Moreover, the comparison between these predicted and known GECs on different external tasks demonstrates the generalization ability of TranscriptionNet.

For reliable predictions, TranscriptionNet must be trained based on multiplex biological networks as most networks have no uniform scale and quality, and one network generally only focuses on certain types of functional relationships. In addition, the complementary effects between the GECs by RNAi, CRISPR, and OE for the same genes are based on the assumption that different types of genetic manipulations on the same genes theoretically should produce similar biological outcomes. However, the fact is that these genetic perturbations are inhomogeneous due to various confounding factors, such as systematic and off-target effects, nonspecific toxicity, and different mechanisms of action [10, 13, 24].

For each target gene, we also investigate the impact of other targets on its prediction performance. We calculate the average or largest of absolute PCC values between the target gene and other target genes to reflect the impact of other target genes or the most related target gene on the specific target gene. Then, we compare these values to the prediction performance metrics of target genes (that is represented by the PCC between a pair of predicted and true GECs of the target gene) in the test set. This relationship can reflect the impact of the target gene’s relevance to other target genes on the prediction performance of its GECs in our model. The results indicate that the performance of predicting GECs of target genes are positively impacted by its relevance to all other target genes or the most related target gene (Supplementary Fig. 9). Especially, we can observe that true GECs of some target genes have larger PCC to its predicted GECs than the average or maximal PCC to other known target genes. For example, the gene PRKRA has an average and largest PCC of 0.856 and 0.965, inferior to the PCC value of 0.974 with its predicted GECs; CHEK2 has an average and largest PCC of 0.842 and 0.962, inferior to the PCC value of 0.968 with its predicted GECs. These results suggest that our model can integrate the relevant information with other target genes to improve the predictive performance.

One of the most important strengths of TranscriptionNet is detecting biological connections between drugs, genes, and diseases. Based on the genome-wide scale transcriptional profiles generated by TranscriptionNet, we can explore biological targets and pathways for drugs and genes associated with pathogenic mechanisms of diseases by directly comparing transcriptional profiles of genetic perturbations and existing high-throughput data and further identify candidate disease treatments with specific molecular mechanisms. Therefore, TranscriptionNet holds promise to not only systemically detect gene function but also enhance drug development and target discovery.

## Methods

The training data for the TranscriptionNet model primarily consist of two components: the first is seven types of gene functional networks, including disease-based gene association network, drug-based gene association network, protein complex–based network, pathway-based gene network, chromosomal location-based gene network, STRING, and protein sequence similarity network. The second component comprises gene expression profiles induced by three types of genetic perturbations: RNAi, OE, and CRISPR-Cas9. The TranscriptionNet model is comprised of two main parts: a Functional network based Deep Neural Network and a Genetic perturbation type-based Self-Attention Network. The detailed processes of data collection and processing, the framework of the TranscriptionNet model, and the handling procedures for the three external generalization tasks are comprehensively described in the Supplementary Text.

**Key Points**

- The TranscriptionNet model is capable of predicting transcription profiles induced by genetic perturbations.
- TranscriptionNet comprises two modules: the Functional network-based deep neural network (FunDNN) and the Genetic perturbation type-based self-attention network (GenSAN).
- FunDNN processes multiple gene functional networks and integrates abundant gene functional association information, while GenSAN utilizes multi-layer Transformer encoder blocks to capture the complementary effects of the same gene across different types of genetic perturbations.

## Supplementary Material

revised_Supplementary_Information_bbae433

Supplementary_Data_1_bbae433

Supplementary_Data_2_bbae433

## Data Availability

RNAi, OE, CRISPR, and compound data can be downloaded from the shared database at https://clue.io./releases/data-dashboard. Network data can be downloaded from the following databases: Disease (https://www.omim.org/), DrugBank (https://www.drugbank.ca/), CORUM (https://mips.helmholtz-muenchen.de/corum/), Pathway Reactome (https://reactome.org), STRING (https://string-db.org), UniProt (https://www.uniprot.org/), chromosomalLocation (http://www.ncbi.nlm.nih.gov/gene), KEGG (https://www.genome.jp/kegg/), and GO_BP (https://www.geneontology.org/). Gene sets for ischemic cardiomyopathy and nonischemic cardiomyopathy can be downloaded from DisGeNET (https://www.disgenet.org/). Transcriptional profiling data for ischemic cardiomyopathy and nonischemic cardiomyopathy model groups can be downloaded from the Gene Expression Omnibus database with accession number GSE46224. In addition to the perturbations in the CMap dataset, GECs generated by RNAi, CRISPR, and OE from all gene members in the input network can be predicted through TranscriptionNet, which can be downloaded from https://github.com/lipi12q/TranscriptionNet/tree/master. The source data of all figures and tables can be found at https://doi.org/10.6084/m9.figshare.25309339.

## Code availability

The code for TranscriptionNet can be found at https://github.com/lipi12q/TranscriptionNet.
