# Supplementary material for: A genome-scale deep learning model to predict gene expression changes of genetic perturbations from multiplex biological networks
Source: Brief Bioinform. 2024 Sep 3;25(5):bbae433. doi: 10.1093/bib/bbae433 (PMC11370636; doi:10.1093/bib/bbae433)
Supplement: revised_Supplementary_Information_bbae433 [file revised_supplementary_information_bbae433.docx]

**Supplementary text**

**Network preprocessing**

To incorporate as much gene functional association information as possible, seven classes of gene interaction networks are curated from different biological repositories. The disease-based gene association network is constructed by connecting two genes related to the same diseases based on the Online Mendelian Inheritance in Man (OMIM), a comprehensive knowledgebase that covers human genes and genetic disorders relationships (<https://www.omim.org/>)[1]. The drug-based gene association network is built by linking two proteins targeted by the same drugs based on known drug-target interactions, which are downloaded from the DrugBank database (<https://www.drugbank.ca/>). The protein complex-based network is created by connecting two genes in the same complex subunits, which are collected from CORUM, a database that provides a manually curated catalog of experimentally characterized protein complexes from mammalian organisms (https://mips.helmholtz-muenchen.de/corum/)[2]. The pathway-based gene network is curated by linking two genes in the same biological pathways, which are downloaded from Reactome, a knowledgebase of biological pathways, reactions, proteins, and molecules (https://reactome.org)[3]. The chromosomal location-based gene network is built by connecting two genes in the same cytogenetic bands, which are curated from Gene, a searchable database of gene-specific contents in the National Center for Biotechnology Information (NCBI) (<http://www.ncbi.nlm.nih.gov/gene>). The five gene networks are constructed based on the assumption that two genes associated with the same biological entities should be more functionally related than two genes associated with different biological entities. The similarity between two genes in these networks is quantified by calculating Jaccard similarity scores. The Search Tool for Recurring Instances of Neighboring Genes (STRING; https://string-db.org) quantitatively integrates different studies and interaction types into a single integrated score for each gene pair based on the total weight of evidence[4]. The protein sequence similarity network is obtained by calculating pairwise Smith–Waterman scores[5]. The sequences of reviewed human proteins are collected from UniProt (https://www.uniprot.org/)[6]. To obtain networks that are comparable in size to other networks, the STRING network is filtered for only the top 10% of interactions by interaction scores, the pathway network keeps gene pairs with a similarity magnitude greater than or equal to 0.2 as edges, and the sequence similarity network retains edges with pairwise similarity scores greater than or equal to 0.23. To unify all networks for analysis, gene names in each network are mapped to human Entrez gene ID using the R package org.Hs.eg.db (version:3.18.0). All these networks cover 26,945 unique human genes and the detailed information of each network is provided in the Supplementary Table 1.

**Gene expression data source and preprocessing**

To build the CMAP dataset, the L1000 platform directly measures 978 landmark genes using Luminex bead-based technology and infers an additional 11,350 genes, of which, 9,196 are well inferred[7]. We download the LEVEL 5 data from the Expanded CMap LINCS Resource 2020 (version: beta) at the LINCS data releases app (https://clue.io/releases/data-dashboard). We focus on the 978 landmark genes of three types of genetic perturbations, including 23,835 shRNA, 142,901 CRISPR, and 34,171 OE treatments. We collate gene targets of genetic perturbagens from the metadata and check the names of all target genes using the R package HGNChelper (version: 0.8.1). In each type of genetic perturbation, we combine all transcriptional profiles for each target gene by the weighted average algorithm, which calculates a weighted average of the gene expression signatures of each target, with coefficients given by a pairwise Spearman correlation matrix between the expression profiles of all signatures[8]. Finally, we obtain unique transcriptional profiles for 8288 genes, corresponding to 4,454 shRNA, 5,319 CRISPR, and 3,538 OE target genes. For each gene in all profiles, we normalize them to [-1, 1] using the MinMax Scaler in Python's scikit-learn (version: 1.3.2), to reduce the differences between genes and accelerate model convergence. In the prediction model of transcriptional profiles, data in each type of genetic perturbation is randomly divided into training, validation, and test sets with a 7:1:2 ratio.

**TranscriptionNet architecture**

TranscriptionNet comprises two stages. The first network that we term FunDNN (**Fun**ctional network based **D**eep **N**eural **N**etwork) takes an array of gene functional networks as inputs to produce pre-transcriptional profiles for RNAi, CRISPR, or OE perturbations. FunDNN contains two main blocks. The first block processes heterogeneous gene networks through a sequence of graph attention network (GAT) layers to learn a unified representation for each gene. Then the integrated gene features are fed into a multi-layer neural network to generate pre-transcriptional profiles for each genetic perturbation. The second network that we term GenSAN (**Gen**etic perturbation type-based **S**elf-**A**ttention **N**etwork) processes pre-transcriptional profiles by axial self-attention framework to capture complementary gene expression information for one type of genetic perturbation from the other two types.

**Functional network-based deep neural network**

Robust and integrated gene representations are learned from various functional networks using the general and scalable deep learning framework for network integration termed BIONIC (Biological Network Integration using Convolutions). This architecture is selected as its encoded features contain substantially more topological and functional information compared to existing architectures[9]. Specifically, each input gene network is represented by its adjacency matrix A where $A_{ij}=A_{ji}=$edge weight value if node *i* and node *j* share an edge and $A_{ij}=A_{ji}=0$ otherwise. BIONIC encodes each input network using three sequential graph attention network (GAT)[10] layers. The gene encoder is described as follows:

$$\mathrm{GAT}\left( A, H \right)= \sigma\left( HW^{T} \right) \left( 1 \right)$$

Where

$$a_{ij}=\frac{A_{ij}exp(\sigma(\partial^{T}[Wh_{i}||Wh_{j}))}{\sum_{K=1} A_{ij}exp(\sigma(\partial^{T}[Wh_{i}||Wh_{j}))} \left( 2 \right)$$

Here, $W$ is the layer-specific trainable weight matrix. $\partial$ is the vector of learnable attention coefficients. $K$ corresponds to nodes in the neighborhood of $i$. $h_{i}$ is the feature vector of node $i$, that is, the $i$th row of feature matrix $H$. The initial feature matrix $H_{init}$is an identity matrix so that each node is uniquely identified. These node features are further mapped to a real-valued dense matrix with a dimension of 2048 through a learned linear transformation. $\sigma$ represents the nonlinear function LeakyReLU. In each GAT layer, the multi-head attention scheme is learned as:

$$\mathrm{GAT}\left( A, H \right)={||}_{k=1}^{K}\sigma\left( a^{\left( k \right)}HW^{\left( k \right)T} \right) (3)$$

where The number of heads $K$=10. After each network is encoded, the network-specific node features are then combined to produce the final unfiled features through a weighted, stochastically masked summation as follows:

$$H_{combined}=\sum_{j=1}^{N} S_{j}m^{(j)}\odot H^{(j)} (4)$$

Here, $N$ is the number of input networks. $S_{j}$ is the learned scaling coefficient for feature representations of network $j$, which enables BIONIC to scale features in a network-wise fashion. All values in $S$ should be positive and sum to 1. $m^{(j)}$ is the node-wise stochastic mask for network $j$, which is designed to randomly drop node feature vectors produced from some networks, forcing the network encoders to learn cross-network dependencies. ⊙ is the element-wise product and $H^{(j)}$ is the learned feature matrix for nodes network $j$.

BIONIC maps $H_{combined}$ to a low-dimensional feature matrix $F$ with a dimension of 512 through a learned linear transformation. In $F$, each row corresponds to a node feature. BIONIC can decode F into reconstructions of the original input networks. To obtain a high-quality F, BIONIC uses an unsupervised training objective to minimize the gap between the reconstructed and the input networks:

$$L_{unsupervised}=\frac{1}{n^{2}}\sum_{j=1}^{N} ||b^{\left( j \right)}\odot(\hat{A}-A^{(j)})\odot b^{(j)}{||}_{F}^{2} (5)$$

Where the reconstructed network $\hat{A}=F\cdot F^{T}$, $n$ is the total number of nodes present in the union of networks, $b^{(j)}$ is a binary mask vector for network $j$ indicating which nodes are present (value of 1) or extended (value of 0) in the network, $A^{(j)}$ is the adjacency matrix for network $j$ and $||\cdot{||}_{F}$ is the Frobenius norm. This loss represents computing the mean squared error between the reconstructed network $\hat{A}$and input $A^{(j)}$ while the mask vectors remove the penalty for reconstructing nodes that are not in the original network $j$ (that is, extended), then summing the error for all networks.

The integrated network features for each gene are standardized using the StandardScaler in Python's scikit-learn to balance the contributions of diverse features and reducing the risk of model bias towards certain features. Then the feature data are further fed into the second block of FunDNN, a multi-layer perceptron (MLP). The MLP model uses multiple dense layers to capture information between different features for each gene described as follows:

$$F^{l+1}=(\sigma\left( W^{l}F^{l}+b^{l} \right))W^{o}+b^{o}$$

Where $\sigma$ is the non-linear activation function, $W^{l}$ and $b^{l}$ are model weights and bias at the l-th layer, $W^{0}$ and $b^{o}$ are model weights and biases at the output layer. Each hidden layer has the same number of nodes while following a dropout layer. The activation function of all layers is LeakyReLu except the penultimate layer is Tanh. The role of the Tanh function is to compress the features in the range of [−1, 1]. The output layer is an innocent linear layer that maps gene features to the predicted transcriptional profiles $\hat{t}$ with a dimension of 978 in the range [−∞, +∞]. To predict transcriptional profiles of genetic perturbations with less discrepancy in the expression of corresponding genes to its true transcriptional profiles, we design a customized loss function PMSE that is a weighted sum of the mean squared error (MSE) and Pearson correlation losses:

$$L_{MSE}=\frac{1}{n}\sum_{i=1}^{n} \left( t_{i}-\hat{t_{i}} \right)^{2} \left( 1 \right)$$

$$L_{Pearson}=\frac{\sum_{i=1}^{n} \left( t_{i}-\bar{t} \right)\left( \hat{t_{i}}-\bar{\hat{t}} \right)}{\sqrt{\sum_{i=1}^{n} \left( t_{i}-\bar{t} \right)^{2}\sqrt{\sum_{i=1}^{n} \left( \hat{t_{i}}-\bar{\hat{t}} \right)^{2}}}} \left( 2 \right)$$

$$\mathrm{PMSE}=\left( 1-\beta\right)L_{MSE}+\beta L_{Pearson} (3)$$

Where $t$ is the true GECs of the corresponding target genes, $\bar{t}$ and $\bar{\hat{t}}$ are the average values of the true and predicted GECs, respectively. $\beta$ is a hyperparameter in the range [0, 1] indicating the relative weight of the two losses. To predict transcriptional profiles for RNAi, CRISPR, or OE perturbations, all weights in the MLP model are separately updated for each type by backpropagation using the Adadelta optimizer. Hyperparameter combinations are automatically chosen for each type of perturbation using Optuna (version: 3.2)[11] and provided in Supplementary Data 2.

**Genetic perturbation type-based self-attention**

Based on the transcriptional profiles predicted by FunDNN (pre-GECs), the second network GenSAN intends to capture complementary information between transcriptional profiles of the three types of genetic perturbations, RNAi, CRISPR, and OE, as well as complementary information between 978 marker genes, through an axial self-attention[12] framework to refine pre-GECs. For example, to refine pre-GECs of RNAi, true GECs of the other two types of perturbations CRISPR and OE for the same gene are loaded together as inputs. The input matrix $\hat{T}_{init}$ has a dimension of $3\times978$. The first row is the pre-GECs of RNAi, and the other two rows are true GECs of CRISPR and OE for the same gene. If lacking true GECs of CRISPR or OE, the corresponding pre-GECs are used.

We use multiple transformer encoder units[13] to effectively learn the interrelated information between transcriptional profiles of the three types of genetic perturbations and 978 marker genes:

$$\hat{T}_{r}^{l}=rowatten\left( \hat{T}^{l}, W_{r}^{l},b_{r}^{l} \right) (1)$$

$$\hat{T}_{c}^{l}=colatten\left( {{(\hat{T}}_{r}^{l})}^{'}, W_{c}^{l},b_{c}^{l} \right) (2)$$

$$\hat{T}^{l+1}=\left( \sigma\left( W_{f}^{l}\hat{T}_{c}^{l}+b_{f}^{l} \right) \right)W^{o}+b^{o} (3)$$

Where each layer $l$ corresponds to a transformer encoder unit and consists of an axial self-attention layer and a feed-forward neural network layer. The row-wise self-attention block establishes attention weights for the transcriptional profiles of the three genetic perturbations, capturing complementary information between them. The column-wise self-attention block allows for the exchange of information between the 978 marker genes. $W_{r}^{l}$ and $b_{r}^{l}$, $W_{c}^{l}$ and $b_{c}^{l}$, $W_{f}^{l}$ and $b_{f}^{l}$ are learnable weight matrices and bias vectors of the row-wise self-attention block, column-wise self-attention block, and feed-forward neural network layer in the $l$th transformer encoder unit.$W^{0}$ and $b^{o}$ are the weights and bias at the output layer of the feed-forward neural network. $\hat{T}^{l}$ is the $l$th hidden transcriptional profile and $\hat{T}^{0}=\hat{T}_{init}$. $\hat{T}_{r}^{l}$, $\hat{T}_{c}^{l}$ are the hidden transcriptional profiles of the $l$th row-wise self-attention block and column-wise self-attention block, respectively.${{(\hat{T}}_{r}^{l})}^{'}$ is the transposed transcription profiles of $\hat{T}_{r}^{l}$, with a dimension of 978×3. σ is the activation function LeakyReLU. Moreover, an iterative refinement termed “recycling” is applied to the attention stack, the resulting matrix is recycled and iteratively updates the input data. Each recycling combines inputs and outputs from the last iteration and produces reinforced outputs with shared weights. The recycling process creates a recurrent network and deepens the whole network without significantly increasing training time and the number of parameters. This has been successfully applied in other areas such as computer vision and protein structure prediction[14, 15].

After processing with attention stacks, the representation of the specific perturbation is extracted from the first row in the output matrix and processed by a multi-layer dense network to predict the transcriptional profile $\hat{t}$. Similar to the prediction of FunDNN, the loss function PMSE is used to train the model.

We use the stochastic gradient descent optimization algorithm to train the model. The hyperparameters of the three types of perturbation models are mostly the same except for the batch size and initial learning rate (Supplementary Table 2). We use Adam with a weight decay of 1e-5, a learning rate warm-up of the first 5 epochs, a linear increase in the learning rate during the prediction phase, and a slow decrease according to the cosine function. After 110 epochs, we stop learning. We use a dropout probability of 0.05 on all layers, and the number of nodes in the hidden layer of the feedforward neural network in each Transformer unit is set to 1024. To avoid neuron death, we use the LeakyReLU activation function instead of the standard ReLu. The learning rate, batch size, number of transformer unit layers, number of attention heads, and number of cycles are manually adjusted throughout the training process, following the tuning method of comparing the three indicators of PCC, MSE, and D on the test set. When the three indicators stop improving, we consider the current hyperparameter combination to be the optimal combination for the model.

All models were trained on an NVIDIA A100 graphics processing unit with 80GB of graphics memory, 128 GB of system memory, and 13 Intel® Xeon® CPUs running at 2.10 GHz.

**Baselines**

The task of predicting transcriptional profiles can be regarded as a multivariate linear regression (MLR) problem. Therefore, the performance of TranscriptionNet is first compared with that of classical MLR models Decision Tree Regression (DTR), K-Nearest neighbors Regression (KNR), Linear Regression (LR), Random Forest Regression (RFR) and eXtreme Gradient Boosting (XGBoost), which have undergone parameter tuning and the resulting optimized hyperparameters are utilized to train each model based on the gene representations learned from BIONIC to predict transcriptional profiles, further statistical validation was conducted using the T-test method to compare the prediction results between TranscriptionNet and the five classical MLR models. Network integration is the primary module for TranscriptionNet. An excellent network integration algorithm should produce accurate and comprehensive gene representations from biological networks. BIONIC used in our model has been proven to outperform existing integration methods across all evaluation types and benchmarks[9]. To further confirm the potency of BIONIC in our experiment, we compare network integration results from BIONIC to three different established integration approaches: a naive union of networks (Union), a deep learning multi-modal autoencoder (deepNF)[16], and a multi-network extension of the node2vec[17] model (multi-node2vec)[18]. The naive union of networks benchmark was created by taking the union of node sets and edge sets across input networks. For edges common to more than one network, the maximum weight was used. deepNF is an integrated framework based on a multimodal deep autoencoder that learns compact, low-dimensional feature representations of proteins from multiple heterogeneous interaction networks. It uses separate network layers to process different types of networks, fuses learned features into a bottleneck layer and finally performs SVM training on the resulting features[19]. Multi-node2vec is an algorithm for multi-network extension of node2vec model, which learns node features from complex multi-layer networks through the Skip-gram neural network model[20]. For methods that produced features (deepNF, multi-node2vec, and BIONIC), a feature dimension of 512 was used to ensure results were comparable across methods. For methods that required a batch size parameter (deepNF and BIONIC), the batch size was set to 2,048 to ensure reasonable computation times. Except for the union method that has no hyperparameters, all other methods use their default hyperparameters. In addition, to ensure the advantage of gene representations encoded from multiple networks over single networks, we compare the network integration results of BIONIC with those using single networks. TranscriptionNet comprises two main stages, FunDNN and GenSAN. In addition, the second network GenSAN contains a “recycling” structure around the truck of the attention stack. We also examine the impact of the recycling process on the performance of TranscriptionNet.

**Coannotation analysis**

We evaluate the quality of predicted GECs by comparing their ability to predict the same functional terms in commonly annotated gene pairs between known GECs and predicted GECs. Here, we calculated the Pearson correlation coefficients between the target genes of known GECs (RNAi: 4454, CRISPR: 5139, OE: 3538) and predicted GECs (RNAi: 22496, CRISPR: 21806, OE: 23427). We obtained the relationship list between different annotation modules and target genes from Online Mendelian Inheritance in Man (OMIM)[1]、DrugBank、CORUM[2]、Pathway Reactome[3]、Kyoto Encyclopedia of Genes and Genomes (KEGG) pathways[21] and GO Biological Processes (BP)[22]databases. The two benchmarks of KEGG and GO Biological Processes (BP) were not used in our model as external benchmarks. For the GO Biological Processes (BP) benchmark, we removed annotation modules mapped to fewer than 20 and more than 500 target genes, leaving 4,203 annotation module-target gene relationship pairs. Comparing the annotation modules of two target genes, we take the two target genes with any common annotation module as the positive set, otherwise as the negative set. Considering the substantial imbalance between the positive set and the negative set, we extract the same number of target gene pairs from the negative set as the positive set, and perform five times of cross-validation. We used the area under the receiver operating characteristic curve (AUROC) and the area under the precision-recall curve (AUPRC) as quantitative standards to assess the ability of known and predicted GECs to predict coannotation genes, respectively.

**Characterization of compound-target interactions**

To investigate the relationship between drugs and protein targets, we downloaded LEVEL 5 from the CMap LINCS resource 2020 (version: beta) containing 720,216 GECs induced by small molecule compounds. We combine all the transcriptomic profiles of each compound using the weighted average algorithm in the L1000 project[8]. Finally, we obtain unique transcriptomic profiles for 33,609 compounds.

We directly calculate the Pearson correlation coefficient between 33,609 compounds and 26,945 genetic perturbation GECs (including known GECs and predicted GECs), resulting in 100 million relationships. Among them, 10,000 pairs have been curated as true drug-target interactions by CMap. We refer to these interactions as the positive set, while keeping all other pairs as the negative set. First, for each type of genetic perturbation, we separately plot the absolute drug-target correlation distribution between the known and predicted GECs in the positive and negative sets. Additionally, to further quantify the performance of GECs in distinguishing different types of drug-target pairs, we calculate the true positive rate and false positive rate, and plot the ROC curves of known and predicted GECs based on different correlation thresholds. Finally, we summarize the correlation using logistic regression analysis for all three types of perturbations and plot the combined ROC curve of the known and predicted GECs.

Considering the data imbalance of this task, we here also calculate the Matthews correlation coefficient (MCC). The results display that both the GECs predicted by TranscriptionNet and the known GECs possess low and similar MCC values (Supplementary Table 5). The results can be attributed to the imbalanced data, and also the biological or technical disparity between the genetic perturbations and chemical drugs targeting the same genes, which have been validated in the L1000 data analysis [7]. Nevertheless, our aim is to evaluate the robustness of the GECs predicted by our model compared with known GECs. We find both predicted and known GECs have similar performance to drug-target interactions, confirming the robustness of the GECs predicted by our model.

**Characterization of disease-gene associations**

To investigate whether the predictive transcriptomic profile can effectively characterize the association between disease and gene, we collected transcriptomic data from the GEO database for human ischemic cardiomyopathy and non-ischemic cardiomyopathy (GEO database accession number: GSE46224)[23]. We use the R package DEseq2 (version: 3.18.0) to analyze the induced gene expression differences for ischemic cardiomyopathy and non-ischemic cardiomyopathy compared to the control group. We extract the 978 marker genes from the differentially expressed gene profiles for the two diseases.

We collected 110 genes associated with ischemic cardiomyopathy and 15 genes associated with non-ischemic cardiomyopathy from the current DisGeNET database (version: 7.0; https://www.disgenet.org/)[24].

We directly calculate the Pearson correlation coefficients between 26,945 genetic perturbation GECs (including known GECs and predicted GECs) and marker genes in the differential expression profiles of diseases. We refer to the genes associated with diseases as the positive set, while all other genes were retained as the negative set. We randomly perform five runs. To quantify the performance of GECs in distinguishing disease genes, we use AUROC and AUPRC to evaluate the ability of known and predicted GECs to predict disease-gene associations, respectively.

**References**

1. Hamosh A, Scott AF, Amberger JS et al. Online Mendelian Inheritance in Man (OMIM), a knowledgebase of human genes and genetic disorders, Nucleic Acids Res 2005;33:D514-517.

2. Giurgiu M, Reinhard J, Brauner B et al. CORUM: the comprehensive resource of mammalian protein complexes-2019, Nucleic Acids Res 2019;47:D559-d563.

3. Gillespie M, Jassal B, Stephan R et al. The reactome pathway knowledgebase 2022, Nucleic Acids Res 2022;50:D687-d692.

4. Snel B, Lehmann G, Bork P et al. STRING: a web-server to retrieve and display the repeatedly occurring neighbourhood of a gene, Nucleic Acids Res 2000;28:3442-3444.

5. Smith TF, Waterman MS. Identification of common molecular subsequences, J Mol Biol 1981;147:195-197.

6. UniProt: the universal protein knowledgebase in 2021, Nucleic Acids Res 2021;49:D480-d489.

7. Subramanian A, Narayan R, Corsello SM et al. A Next Generation Connectivity Map: L1000 Platform and the First 1,000,000 Profiles, Cell 2017;171:1437-1452.e1417.

8. Smith I, Greenside PG, Natoli T et al. Evaluation of RNAi and CRISPR technologies by large-scale gene expression profiling in the Connectivity Map, PLoS Biol 2017;15:e2003213.

9. Forster DT, Li SC, Yashiroda Y et al. BIONIC: biological network integration using convolutions, Nat Methods 2022;19:1250-1261.

10. Velickovic P, Cucurull G, Casanova A et al. Graph Attention Networks 2017;abs/1710.10903.

11. Akiba T, Sano S, Yanase T et al. Optuna: A Next-generation Hyperparameter Optimization Framework 2019.

12. Huang Z, Wang X, Wei Y et al. CCNet: Criss-Cross Attention for Semantic Segmentation, IEEE Trans Pattern Anal Mach Intell 2023;45:6896-6908.

13. Vaswani A, Shazeer NM, Parmar N et al. Attention is All you Need. In: Neural Information Processing Systems. 2017.

14. Carreira J, Agrawal P, Fragkiadaki K et al. Human Pose Estimation with Iterative Error Feedback 2015:4733-4742.

15. Jumper J, Evans R, Pritzel A et al. Highly accurate protein structure prediction with AlphaFold, Nature 2021;596:583-589.

16. Gligorijevic V, Barot M, Bonneau R. deepNF: deep network fusion for protein function prediction, Bioinformatics 2018;34:3873-3881.

17. Grover A, Leskovec J. node2vec: Scalable Feature Learning for Networks, Kdd 2016;2016:855-864.

18. Wilson JD, Baybay M, Sankar R et al. Analysis of population functional connectivity data via multilayer network embeddings 2018;9:99 - 122.

19. Awad M, Khanna R. Support Vector Machines for Classification. Efficient Learning Machines: Theories, Concepts, and Applications for Engineers and System Designers. Berkeley, CA: Apress, 2015, 39-66.

20. Brazma A, Hingamp P, Quackenbush J et al. Minimum information about a microarray experiment (MIAME)-toward standards for microarray data, Nat Genet 2001;29:365-371.

21. Kanehisa M, Goto S. KEGG: kyoto encyclopedia of genes and genomes, Nucleic Acids Res 2000;28:27-30.

22. Ashburner M, Ball CA, Blake JA et al. Gene ontology: tool for the unification of biology. The Gene Ontology Consortium, Nat Genet 2000;25:25-29.

23. Yang KC, Yamada KA, Patel AY et al. Deep RNA sequencing reveals dynamic regulation of myocardial noncoding RNAs in failing human heart and remodeling with mechanical circulatory support, Circulation 2014;129:1009-1021.

24. Piñero J, Saüch J, Sanz F et al. The DisGeNET cytoscape app: Exploring and visualizing disease genomics data, Comput Struct Biotechnol J 2021;19:2960-2967.


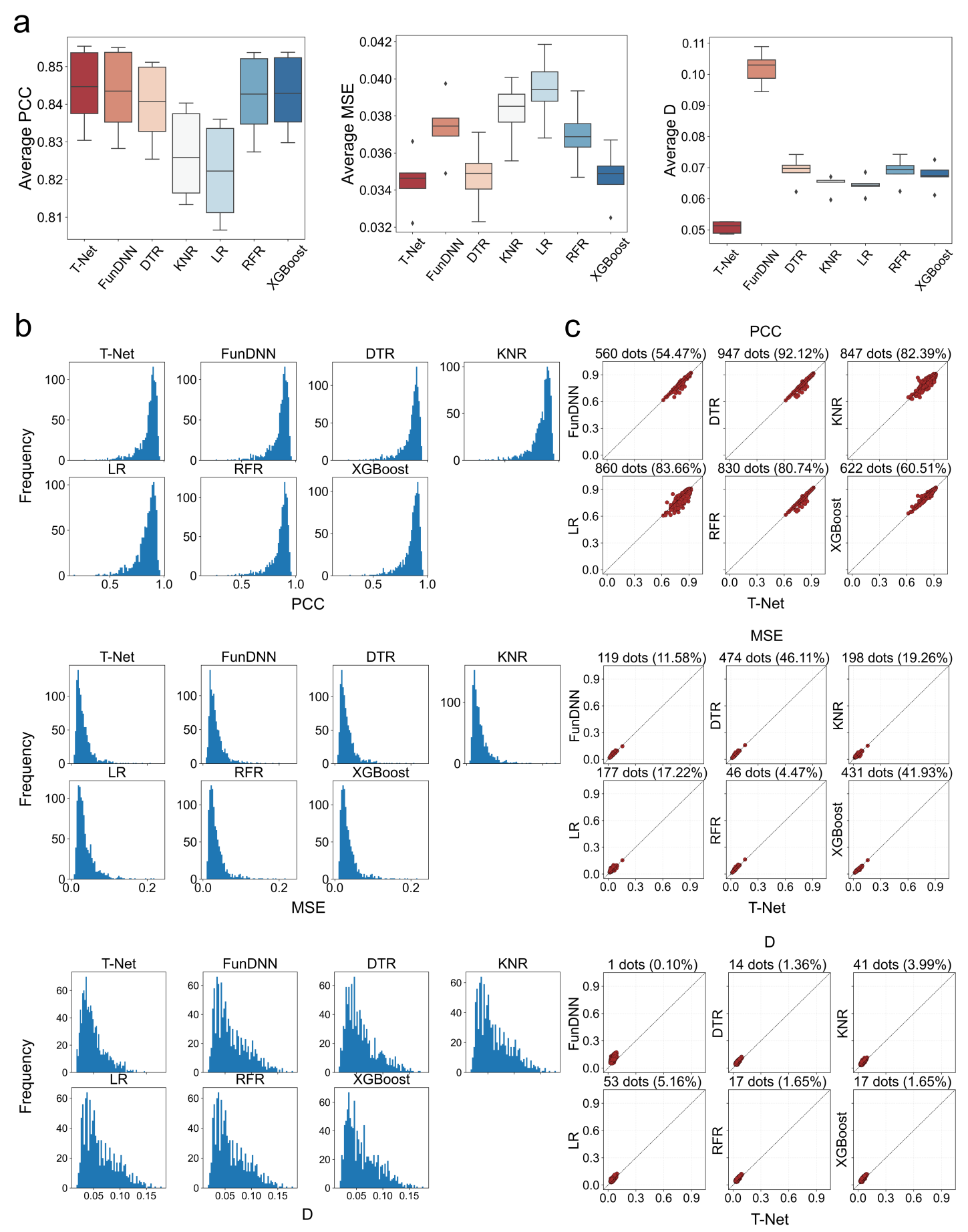


**Supplementary Figure.1** | **Comparison of TranscriptionNet to baseline models for predicting GECs of** **CRISPR**. **a.** Box plots for three metrics (Pearson correlation coefficients (PCC), Mean Square Error (MSE), Kolmogorov-Smirnov (KS) test statistic maximum distance (D)) on the test set for six models: TranscriptionNet, FunDNN, DTR, KNR, LR, RFR and XGBoost. **b.** Distribution of PCC, MSE, and D for the seven models on the test dataset. Data are obtained from five random runs. **c.** The profile-wise comparative analysis of PCC, MSE, and D in the test dataset between TranscriptionNet and the other six models. The x-axis represents the results predicted by the TranscriptionNet model, while the y-axis represents the results predicted by the other five models. The dots below the diagonal indicate that TranscriptionNet has a higher PCC value and lower MSE and D values compared to other models.


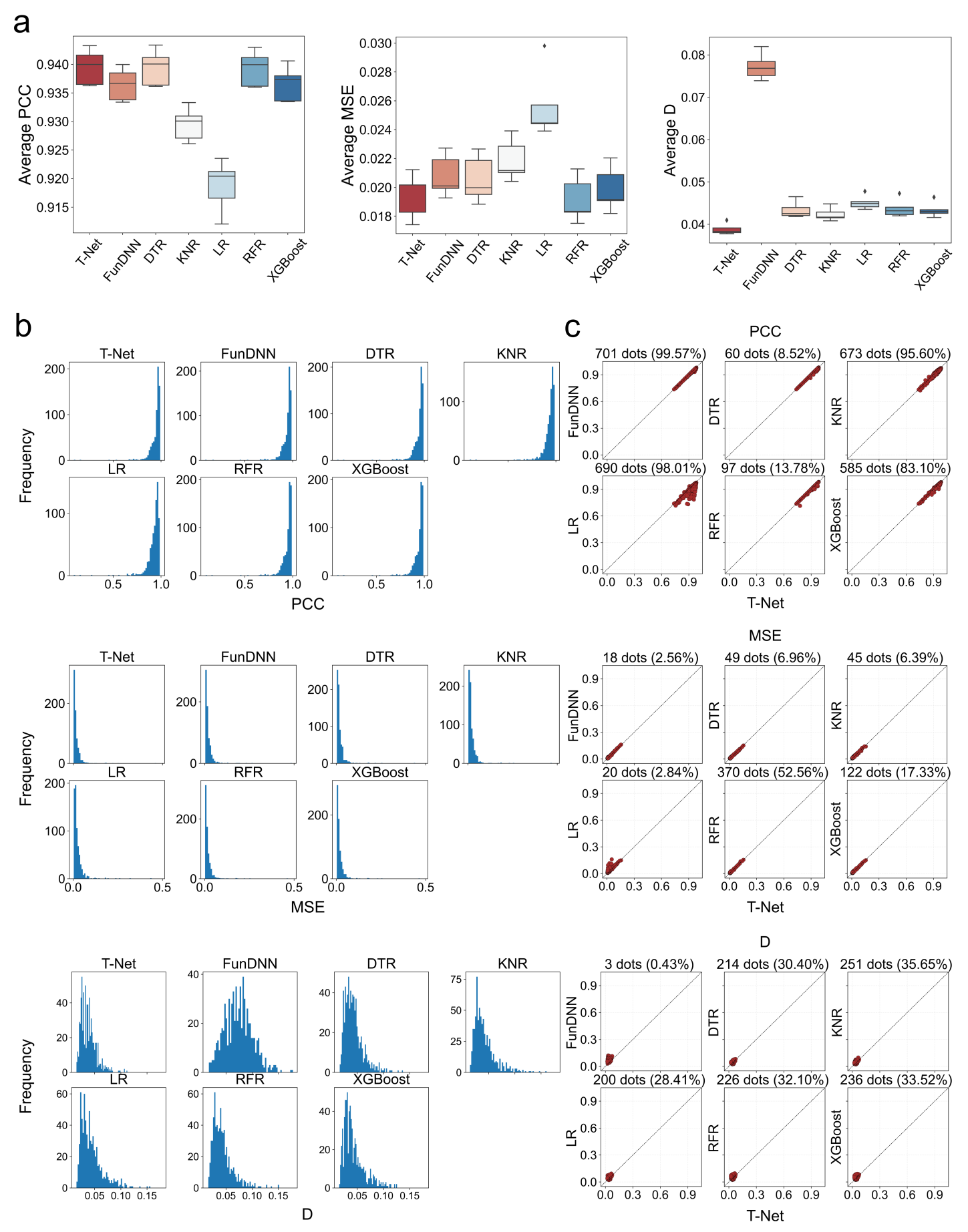


**Supplementary Figure 2** | **Comparison of TranscriptionNet to baseline models for predicting GECs of** **OE**. **a.** Box plots for three metrics (Pearson correlation coefficients (PCC), Mean Square Error (MSE), Kolmogorov-Smirnov (KS) test statistic maximum distance (D)) on the test set for six models: TranscriptionNet, FunDNN, DTR, KNR, LR, RFR and XGBoost. **b.** Distribution of PCC, MSE, and D for the seven models on the test dataset. Data are obtained from five random runs. **c.** The profile-wise comparative analysis of PCC, MSE, and D in the test dataset between TranscriptionNet and the other six models. The x-axis represents the results predicted by the TranscriptionNet model, while the y-axis represents the results predicted by the other five models. The dots below the diagonal indicate that TranscriptionNet has a higher PCC value and lower MSE and D values compared to other models.


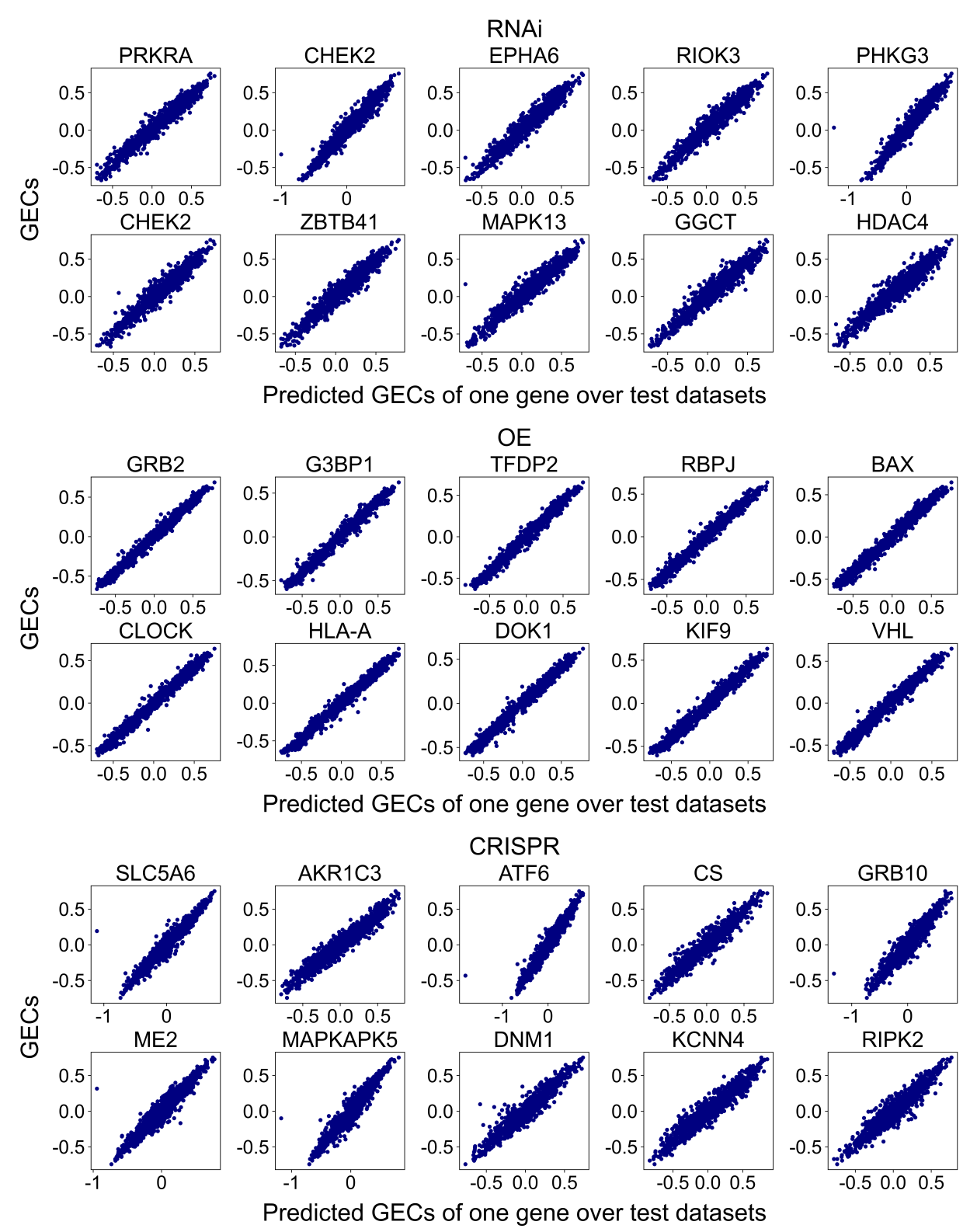


**Supplementary Figure 3** | Density scatter plots of the true GECs versus the predicted GECs for top ten target genes in the test dataset for three types of genetic perturbations including RNAi, OE and CRISPR. In the RNAi experiment, the top ten genes include PRKRA, CHEK2, EPHA6, RIOK3, PHKG3, CHEK2, ZBTB41, MAPK13, GGCT and HDAC4. In the OE experiment, the top ten genes include GRB2, G3BP1, TFDP2, RBPJ, BAX, CLOCK, HLA-A, DOK1, KIF9 and VHL. In the CRISPR experiment, the top ten genes include SLC5A6, AKR1C3, ATF6, CS, GRB10, ME2, MAPKAPK5, DNM1, KCNN4 and RIPK2.


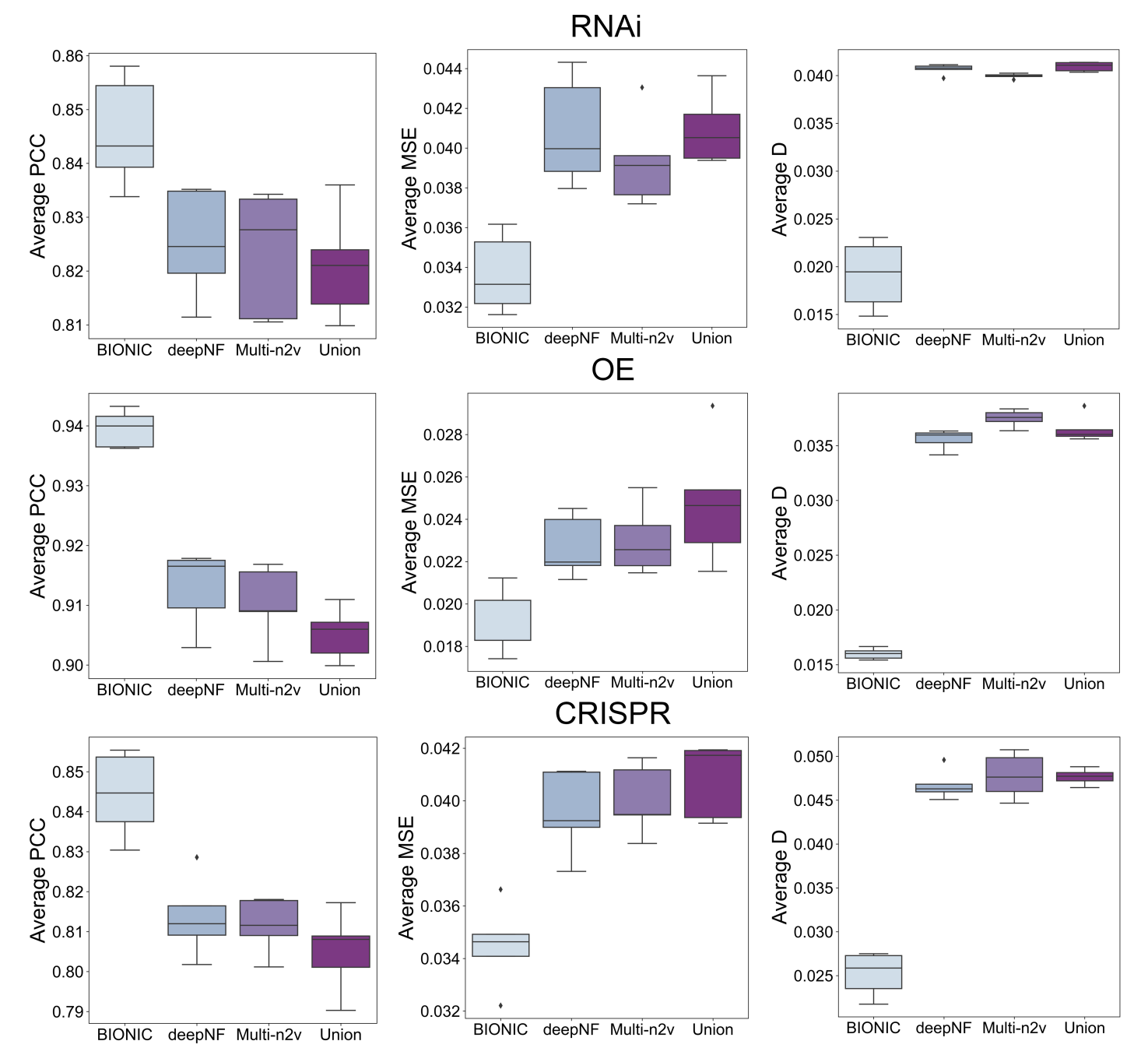


**Supplementary Figure 4** | **Comparison of network integration methods.** Box plots of four integration methods (BIONIC, deepNF, Multi-node2vec, and Union) on three metrics (Pearson correlation coefficients (PCC), Mean Square Error (MSE), Kolmogorov-Smirnov (KS) test statistic maximum distance (D)) on the test dataset. Data are obtained from five random runs.


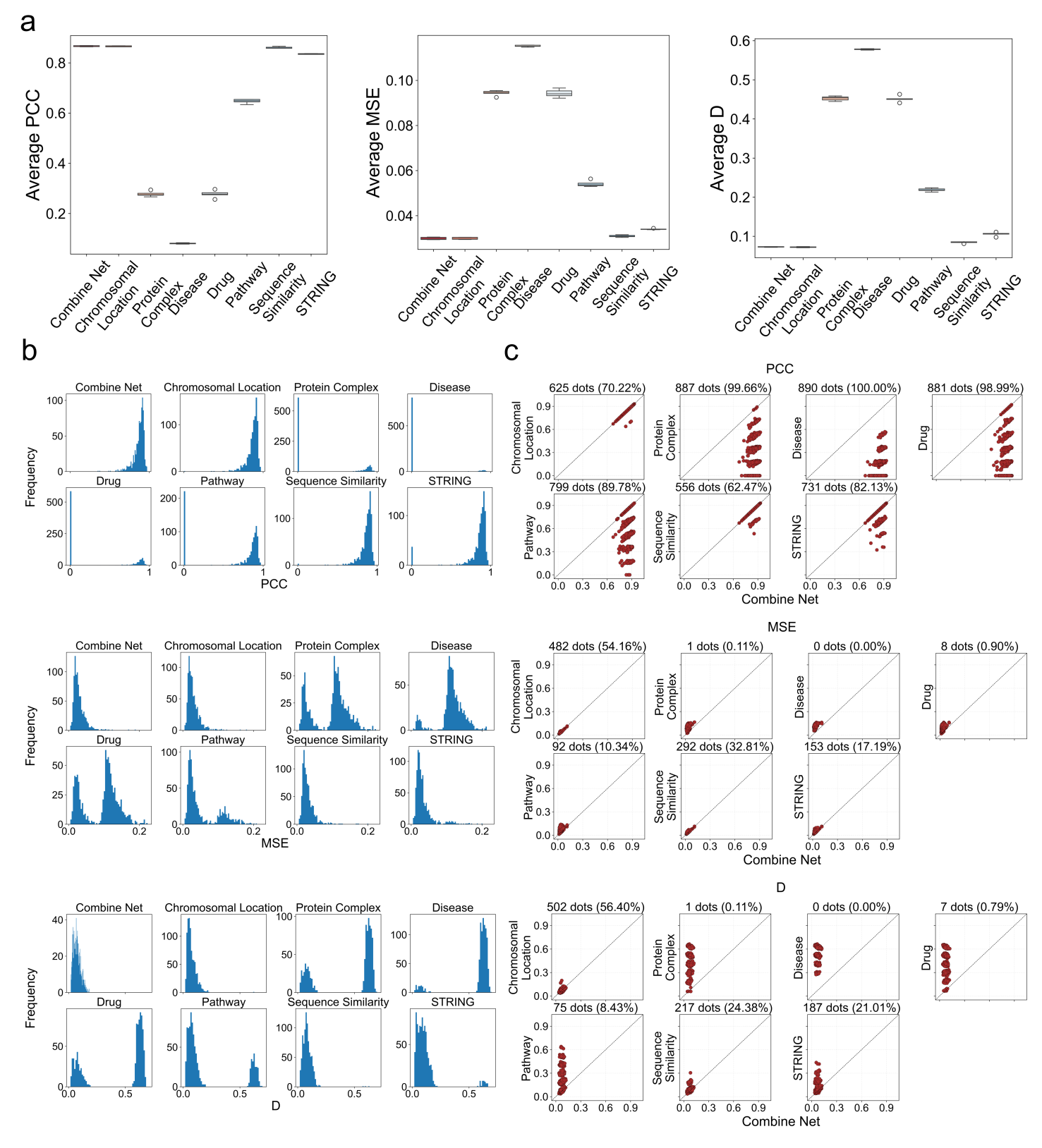


**Supplementary Figure.5** | **Comparison of multiple networks and individual networks for predicting GECs of RNAi based on the TranscriptionNet model.** **a.** Box plots for three metrics (Pearson correlation coefficient (PCC), Mean Square Error (MSE), Kolmogorov-Smirnov (KS) test statistic maximum distance (D)) on the test dataset for eight networks: multiple networks, the chromosomal location-based gene network, the protein complex based network, the disease-based gene association network, the drug-based gene association network, the pathway-based gene network, the protein sequence similarity network, and the STRING protein-protein interaction network. **b.** Distribution of PCC, MSE, and D for each of the eight networks on the test dataset. Data are obtained from five random runs. **c.** The profile-wise comparative analysis of PCC, MSE, and D between multiple networks and other seven single networks. The x-axis represents the results predicted by multiple networks, while the y-axis represents the results predicted by other single networks. The dots below the diagonal indicate that multiple networks have a higher PCC value and lower MSE and D values compared to the other networks.


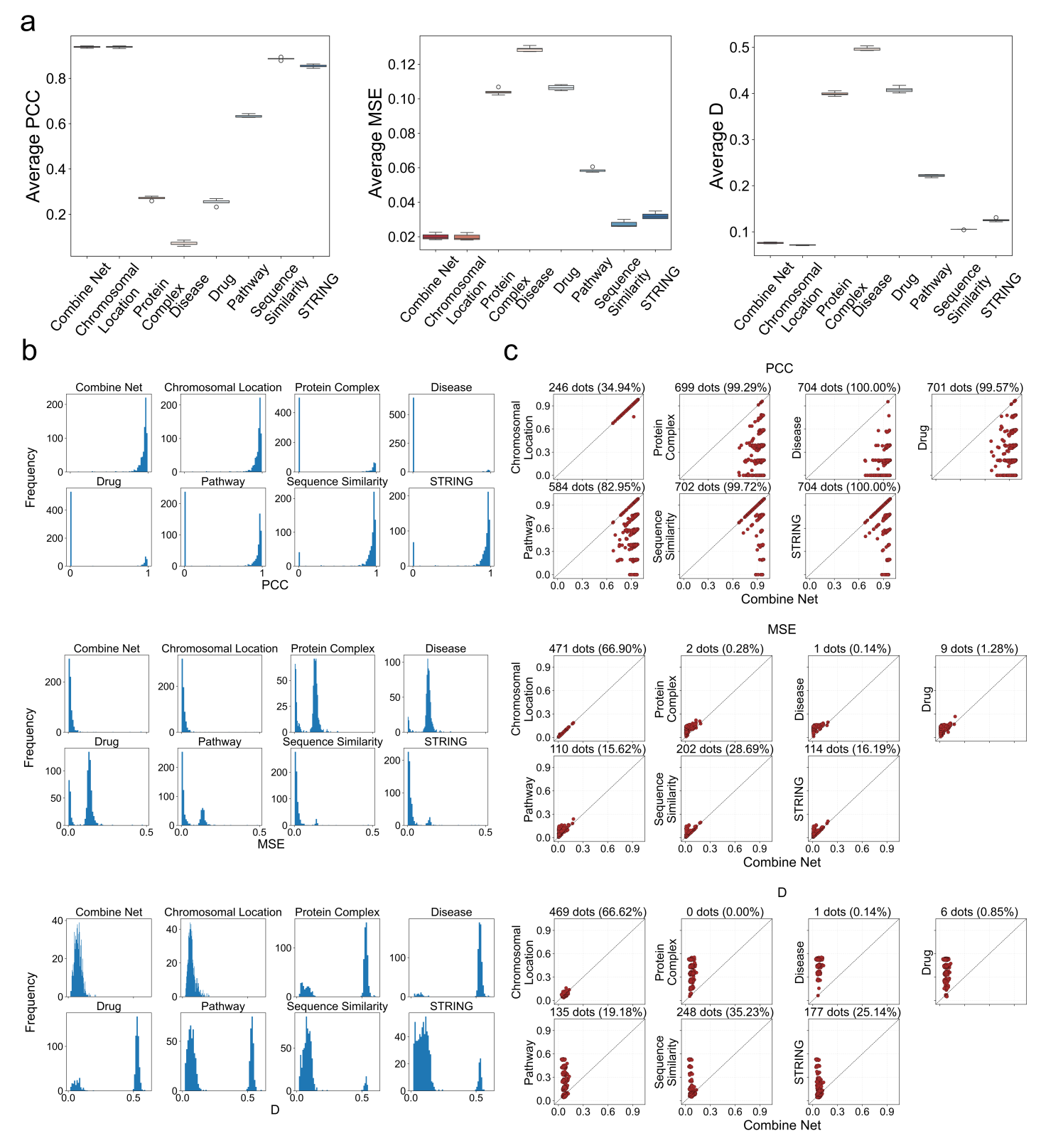


**Supplementary Figure.6** | **Comparison of multiple networks and individual networks for predicting GECs of OE based on the TranscriptionNet model.** **a.** Box plots for three metrics (Pearson correlation coefficient (PCC), Mean Square Error (MSE), Kolmogorov-Smirnov (KS) test statistic maximum distance (D)) on the test dataset for eight networks: multiple networks, the chromosomal location-based gene network, the protein complex based network, the disease-based gene association network, the drug-based gene association network, the pathway-based gene network, the protein sequence similarity network and the STRING protein-protein interaction network. **b.** Distribution of PCC, MSE, and D for each of the eight networks on the test dataset. Data are obtained from five random runs. **c.** The profile-wise comparative analysis of PCC, MSE, and D between multiple networks and other seven single networks. The x-axis represents the results predicted by multiple networks, while the y-axis represents the results predicted by other single networks. The dots below the diagonal indicate that multiple networks have a higher PCC value and lower MSE and D values compared to the other networks.


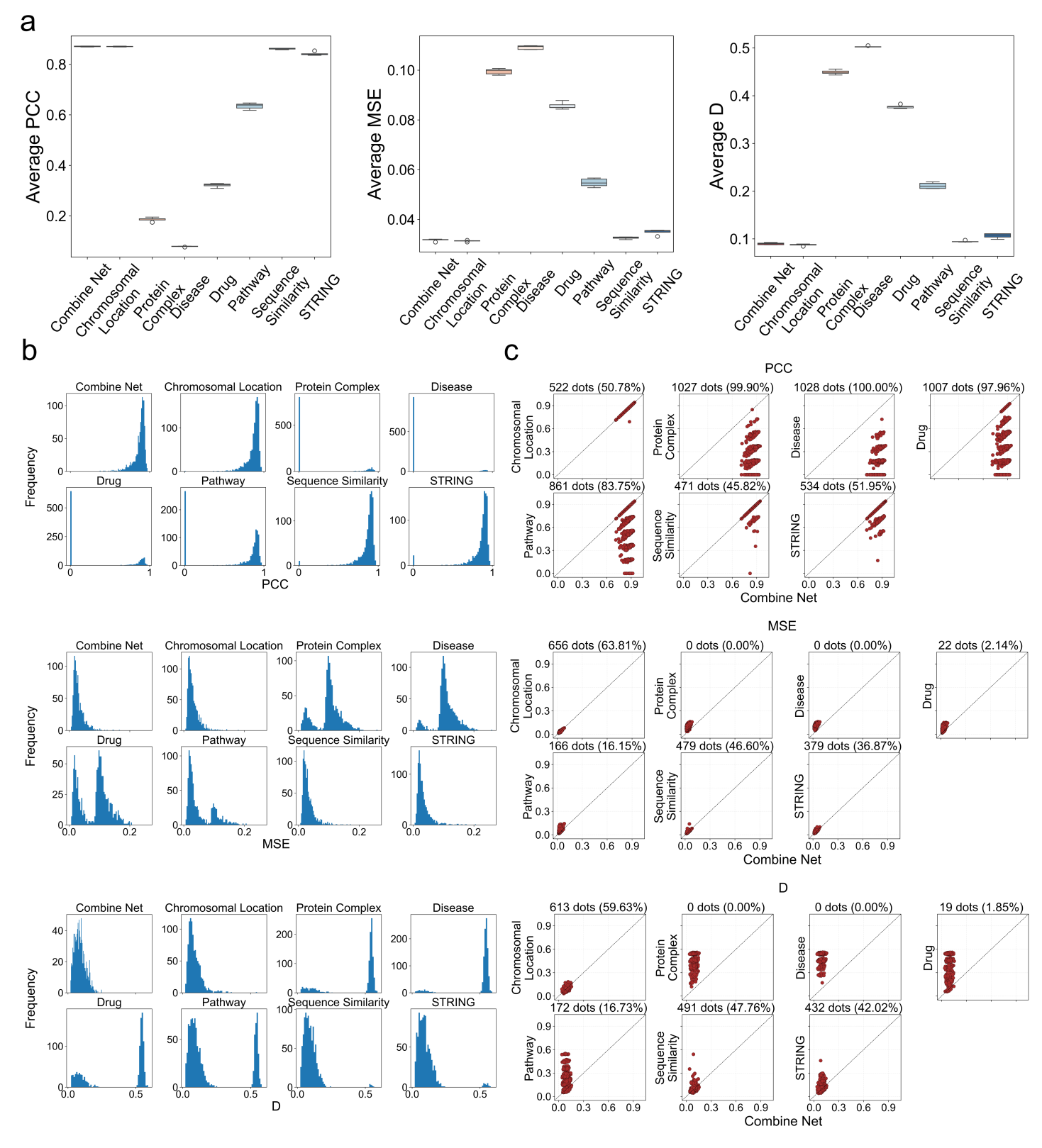


**Supplementary Figure.7 | Comparison of multiple networks and individual networks for predicting GECs of CRISPR** **based on the TranscriptionNet model.** **a.** Box plots for three metrics (Pearson correlation coefficient (PCC), Mean Square Error (MSE), Kolmogorov-Smirnov (KS) test statistic maximum distance (D)) on the test dataset for eight networks: multiple networks, the chromosomal location-based gene network, the protein complex based network, the disease-based gene association network, the drug-based gene association network, the pathway-based gene network, the protein sequence similarity network and the STRING protein-protein interaction network. **b.** Distribution of PCC, MSE, and D for each of the eight networks on the test dataset. Data are obtained from five random runs. **c.** The profile-wise comparative analysis of PCC, MSE, and D between multiple networks and other seven single networks. The x-axis represents the results predicted by multiple networks, while the y-axis represents the results predicted by other single networks. The dots below the diagonal indicate that multiple networks have a higher PCC value and lower MSE and D values compared to the other networks.


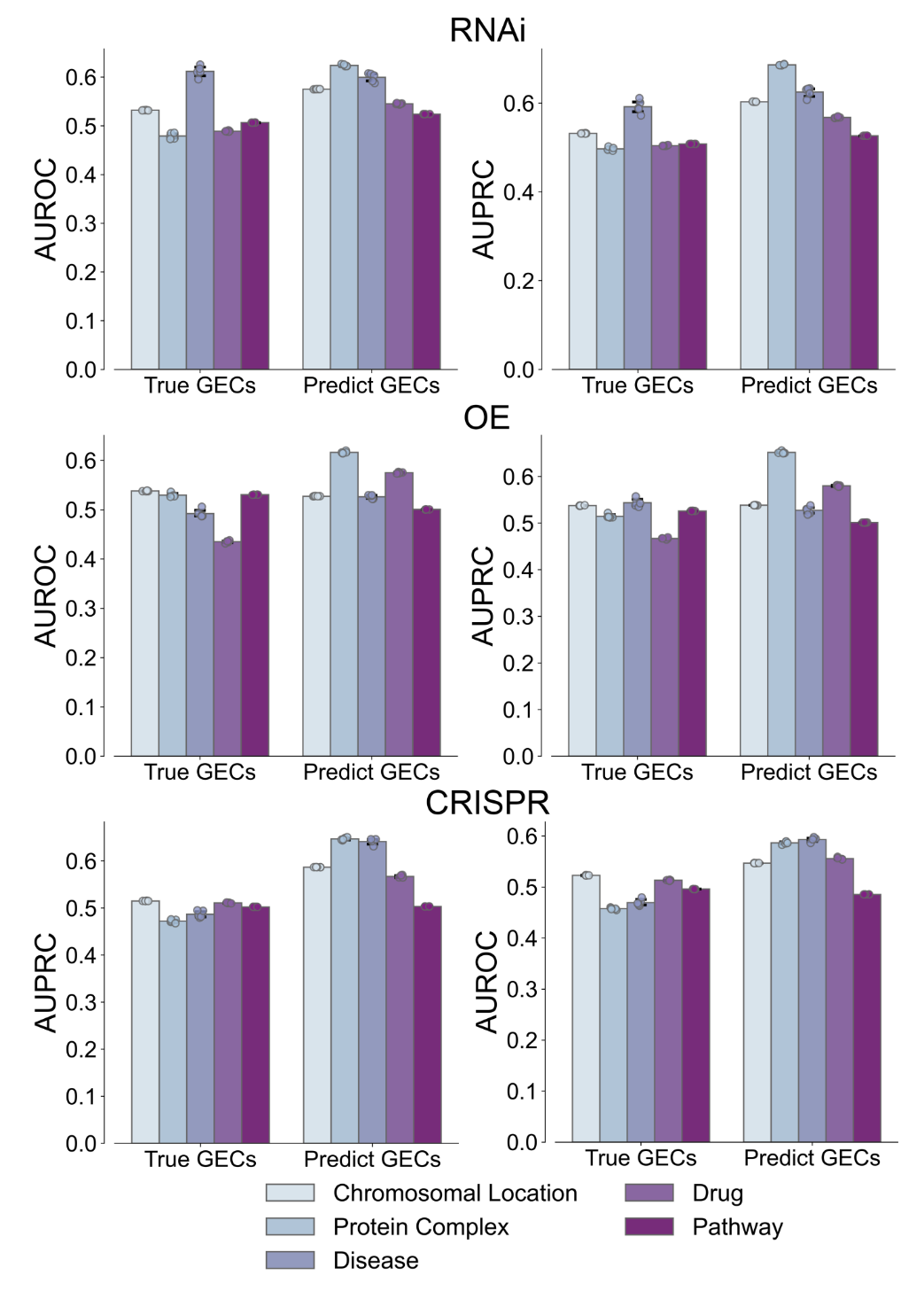


**Supplementary Figure 8** | **Gene function characterization.** We perform gene coannotation analysis based on the true and predicted GECs in five functional networks (the chromosomal location-based gene network, the protein complex-based network, the disease-based gene association network, the drug-based gene association network, and the pathway-based gene network). The statistics are carried out over five random runs. The evaluation criteria include the area under the receiver operating characteristic curve (AUROC) and the area under the precision-recall curve (AUPRC). The data are represented as the average value, with error bars indicating the 95% confidence interval of 5 independent samples, and floating points representing the accurate values of 5 independent samples.


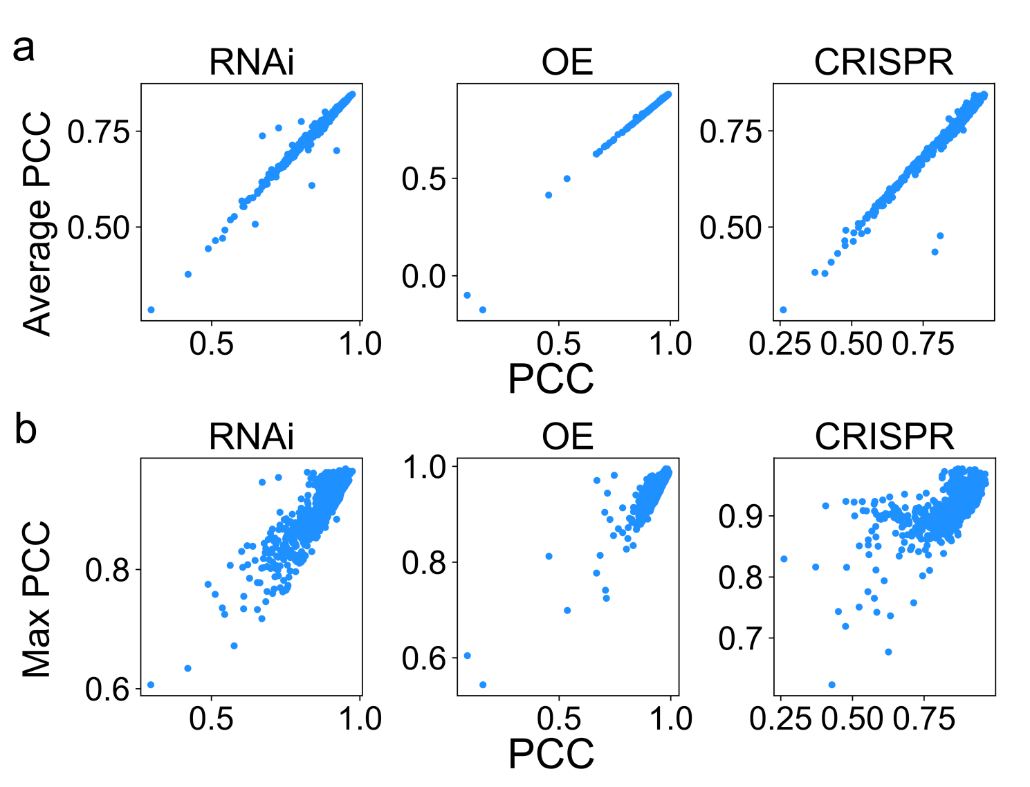


**Supplementary Figure 9 | The impact of other targets on its prediction performance.** **a.** scatter plot between the performance metrics (that is represented by the PCC between pair of predicted and true GECs of the target gene) and the characteristics of the target gene in the PCC distribution (that is represented by the average of absolute PCC values between the target gene and other target genes). **b.** scatter plot between the performance metrics and the characteristics of the target gene in the PCC distribution (that is represented by the maximal absolute PCC values between the target gene and other target genes).

**Supplementary Table 1** detailed information of gene networks

| **Network** | **Interaction type** | **Node** | **Edge** | **Database** | **Data source** |
| --- | --- | --- | --- | --- | --- |
| the disease-based gene association network | Disease genes | 995 | 4047 | OMIM | https://www.omim.org/ |
| the drug-based gene association network | Drug targets | 2792 | 131193 | DrugBank | https://www.drugbank.ca/ |
| the protein complex-based network | Genes in protein complex | 3407 | 40170 | CORUM | https://mips.helmholtz-muenchen.de/corum/ |
| the pathway-based gene network | Genes in pathways | 10623 | 1787207 | Reactome | https://reactome.org |
| the STRING protein-protein interaction network | Co-  Expression | 17844 | 535462 | STRING | https://string-db.org |
| protein sequence similarity network | Sequence  similarity | 18586 | 4156924 | UniProt | <https://www.uniprot>  .org/ |
| the chromosomal location-based gene network | The positions of cytogenetic bands with a chromosome | 26813 | 860164 | NCBI | <http://www.ncbi.nlm>  .nih.gov/gene |

**Supplementary Table 2** detailed information of GenSAN model hyperparameters

| Sub-Model | RNAi | OE | CRISPR |
| --- | --- | --- | --- |
| Training epoch | 110 | 110 | 110 |
| Warm-up epoch | 5 | 5 | 5 |
| Learning rate | 4.5·10^-6^ | 3.6·10^-6^ | 8·10^-6^ |
| Dropout rate | 0.05 | 0.05 | 0.05 |
| Batch size | 32 | 16 | 32 |
| Hidden size | 1024 | 1024 | 1024 |
| Number layer | 3 | 3 | 3 |
| Number head | 2 | 2 | 2 |
| Recycle times | 3 | 3 | 3 |
| Loss beta | 0.1 | 0.1 | 0.1 |
| Weight decay | 1e-5 | 1e-5 | 1e-5 |

**Supplementary Table 3** detailed information of MLR model hyperparameters

| MLR | Hyperparameters |
| --- | --- |
| DTR | criterion="friedman_mse", splitter="best", max_features=None, max_depth=2, min_samples_split=2, min_samples_leaf=1, random_state=42 |
| KNR | n_neighbors=7, weights="uniform", algorithm="auto", leaf_size=15, p=2, metric="minkowski" |
| LR | fit_intercept=False, copy_X=False, n_jobs=None, positive=False |
| RFR | n_estimators=150, criterion="squared_error", max_depth=2, min_samples_split=2, min_samples_leaf=1, max_features=1.0, random_state=42 |
| XGBoost | max_depth=3, learning_rate=0.1, n_estimators=100, objective="reg:linear", booster="gbtree", gamma=0, min_child_weight=1, subsample=1, colsample_bytree=1, reg_alpha=0, random_state=42 |

**Supplementary Table 4** statistical significance between TranscriptionNet and MLR models

|  | RNAi | | | OE | | | CRISPR | | |
| --- | --- | --- | --- | --- | --- | --- | --- | --- | --- |
|  | PCC | MSE | D | PCC | MSE | D | PCC | MSE | D |
| FunDNN | 0.967209 | 0.056903 | 8.34E-08 | 0.14461 | 0.111654 | 5.78E-12 | 0.871992 | 0.026037 | 1.32E-07 |
| DTR | 0.454585 | 0.957549 | 1.90E-08 | 0.9634 | 0.176798 | 2.03E-08 | 0.541084 | 0.809802 | 4.39E-07 |
| KNR | 0.013761 | 0.008361 | 7.60E-07 | 0.000773 | 0.019046 | 0.000436 | 0.039833 | 0.007584 | 1.31E-05 |
| LR | 0.002307 | 0.000537 | 4.53E-06 | 2.88E-05 | 0.000908 | 0.104786 | 0.017766 | 0.00199 | 9.00E-05 |
| RFR | 0.953852 | 0.580032 | 3.08E-08 | 0.896201 | 0.955466 | 2.52E-08 | 0.756377 | 0.045786 | 3.50E-07 |
| XGBoost | 0.583175 | 0.588441 | 8.74E-08 | 0.172096 | 0.444277 | 5.19E-08 | 0.826885 | 0.811911 | 2.27E-06 |

**Supplementary Table 5** The MCC values for characterizing drug-target interactions based on the predicted GECs by TranscriptionNet and the known GECs of three types of genetic perturbations.

| Genetic perturbation | MCC based on known GECs | MCC based on predicted GECs |
| --- | --- | --- |
| RNAi | 0.178 | 0.163 |
| OE | 0.232 | 0.229 |
| CRISPR | 0.154 | 0.120 |
